# Evaluation and Characterization of Functionally Graded Adhesive Joints: Experimental and Numerical Analyses

**DOI:** 10.3390/polym16243561

**Published:** 2024-12-20

**Authors:** Yanan Zhang, Pedro Gálvez, Miguel Angel Martínez, Juana Abenojar, Magd Abdel Wahab

**Affiliations:** 1 Soete Laboratory, Faculty of Engineering and Architecture, Ghent University, B-9052 Ghent, Belgium; yanan.zhang@ugent.be; 2 Sika S.A.U., Alcobendas, 28108 Madrid, Spain; galvez.pedro@ch.sika.com; 3 Materials Science and Engineering Department, Universidad Carlos III de Madrid, 28911 Leganés, Spain; mamc@ing.uc3m.es; 4 College of Engineering, Yuan Ze University, Taoyuan 32003, Taiwan; 5 Parallel and Distributed Systems Laboratory, Jožef Stefan Institute, Jamova Cesta 39, SI-1000 Ljubljana, Slovenia

**Keywords:** epoxy resin, graduated joint, curing process, finite element method, single lap joints

## Abstract

Epoxy resins have exhibited exceptional performance in engineering applications, particularly as a replacement for traditional mechanical joints in adhesive bonding. This study evaluates the suitability of two innovative adhesives, SikaPower^®^-1511 and SikaPower^®^-1548, in various graded configurations. The thermal curing behavior of the adhesives was analyzed using differential scanning calorimetry (DSC) and Fourier transform infrared spectroscopy (FTIR). Shear tests and finite element simulations were employed to investigate the strength performance and interfacial stress distribution of four adhesive configurations, including single and graded joints in single lap adhesive joints. The results show that SikaPower^®^-1548 reveals a slower heat-curing rate and achieves an average shear limit load of 9 MPa, outperforming the more rigid SikaPower^®^-1511, which reaches 4 MPa. Ultimate load predictions indicate that the shear strength of the 1511-1548-1511 graded configuration is slightly lower than that of SikaPower^®^-1511, with a decrease of 8.86%. In contrast, the 1548-1511-1548 configuration demonstrates a significant improvement, achieving a 32.20% increase in shear strength, along with a 13.12% reduction in peel stress field intensity at the interface end and a 12.21% reduction in shear stress field intensity. Overall, the experimental and simulation results highlight the significant advantages of graded joints over traditional single joints in alleviating stress concentrations and enhancing joint strength. Additionally, the research confirms the potential of epoxy resins in advanced engineering applications, providing a reliable theoretical foundation and technical guidance for the design of graded adhesives.

## 1. Introduction

Adhesive bonding is among the most widely employed techniques for joint structures [1,2,3]. Compared to traditional connection methods such as mechanical fastening and welding, it offers several advantages, including reduced structural weight and the elimination of stress concentrations caused by holes in mechanical connections or solder joints in welding [4,5]. Such characteristics make adhesive bonding an ideal solution to meet the increasing demand for lightweight designs. Epoxy resin, as one of the most important and widely used thermosetting polymers in various adhesive materials, exhibits excellent mechanical properties, such as high tensile, flexural, compressive and fatigue strengths, as well as excellent adhesion, low shrinkage after curing, good corrosion resistance and excellent thermal properties [6,7]. These properties make epoxy resin widely used in protective coatings [8,9], adhesives [10,11] and composite materials [12,13,14]. Epoxy resin adhesives are currently used in various fields such as aerospace, automotive, industrial manufacturing, construction, civil engineering, and general manufacturing [15,16,17].

Epoxy resin is a thermosetting polymer characterized by the presence of epoxy groups (oxirane groups) in its molecular structure [18]. These groups undergo curing reactions to form a three-dimensional crosslinked network, whose density critically influences the resin’s performance [19]. Curing temperature, time, and conditions play a vital role in determining adhesive properties and bonding strength [20]. Therefore, understanding the curing system of epoxy resins is essential for optimizing the design and performance of adhesive joints [21]. In general, the curing process can be categorized into three stages: initial curing, basic curing, and post-curing [2]. Currently, researchers have developed various techniques to characterize the curing kinetics of epoxy resins [22,23,24,25].

Differential scanning calorimetry (DSC) operates on the principle that the heat released during the exothermic reaction of a thermosetting material is proportional to its curing rate. The exothermic curve obtained from the curing process provides key parameters, including the curing onset, curing rate, peak temperature, and enthalpy change (ΔH). These parameters are further utilized to calculate the degree of conversion (α) and activation energy (E_a_). The glass transition temperature (T_g_), which indicates the degree of cross-linking in epoxy resin chains during the curing process, also served as a key thermodynamic parameter for evaluating the thermal stability of materials and can be determined using DSC. The measurement methods of DSC include dynamic [20] and isothermal scans.

Fourier transform infrared spectroscopy (FTIR) represents another commonly used technique for analyzing the curing kinetics of thermosetting epoxy resins, offering distinct advantages [26]. By examining the absorption peaks in FTIR spectra, the chemical structure of the material can be characterized. Furthermore, the curing process can be directly investigated by monitoring the concentration variations of different functional groups within the resin system during curing.

Currently, these approaches are extensively employed to investigate the thermal curing behavior and mechanical properties of epoxy resin materials [27,28,29,30]. Şengül et al. [31] used FTIR to investigate the curing reaction of epoxy resin adhesives, focusing on the interaction between the resin, curing agent, and various additives and fillers. Zhang et al. [32] established the curing kinetic equation of epoxy asphalt through DSC analysis at different heating rates, providing insights into the curing reaction rate and process. Additionally, the curing mechanism was investigated using FTIR spectroscopy and in-situ fluorescence microscopy. Duller et al. [33] investigated the curing behavior of sorbitol-modified melamine-formaldehyde (MF) resins using DSC and FTIR spectroscopy. Variations in E_a_ during curing highlighted the complex reaction kinetics, while FTIR analysis revealed altered and enhanced cross-linking mechanisms in the modified resins. Jouenne et al. [34] employed DSC to investigate the impact of flax fiber moisture content on the curing process of epoxy resin revealing that water in wet fibers significantly accelerates the curing reaction by increasing entropy change, resulting in higher E_a_. In the research of YÜKsel et al. [35], DSC, FTIR, dynamic mechanical analysis (DMA), and thermogravimetric analysis (TGA) were utilized to analyze the chemical, mechanical, and thermal properties of polyurethane-epoxy resin materials. Singh et al. [36] used FTIR and DSC to study the glass transition temperature (T_g_) and the enhancement of mechanical and thermal properties in epoxy resins modified with polydimethylsiloxane (PDMS) for advanced adhesive applications. Aliakbari et al. [37] employed transmission electron microscopy (TEM), FTIR, and DSC to investigate the effect of varying amounts of emulsion latex (containing core-shell rubber particles) on the mechanical properties, thermal stability, and single-lap shear strength of cured epoxy resin adhesives.

Failure strength tests, such as tensile tests, are commonly conducted to evaluate the inherent mechanical properties of adhesives. For epoxy resin adhesive joints, adhesion strength and adhesion toughness are typically assessed using the lap shear test [38,39]. However, the strength of adhesive joints is determined not only by the inherent mechanical properties of the adhesive material but also by a combination of other critical factors, including the quality of the surface treatment [40], the geometry of the joint [41,42], the thickness of the adhesive layer, the nature and magnitude of external loading, environmental influences [43], etc.

Mixed adhesives and functionally graded adhesives, which allow variations in mechanical properties across the overlap region, are effective strategies for enhancing the strength of adhesive structures [44,45]. Due to the uneven stress distribution along the bonding line, significant stress singularities occur at the adhesive ends often leading to joint failure [46]. To address this, a tough adhesive with a lower elastic modulus is applied at the ends of the overlap region, while a rigid adhesive with a higher elastic modulus is positioned in the central region. This arrangement effectively mitigates the edge effect of the joint, reducing the concentration of peel stress and shear stress in critical areas, and thereby improving the structural strength of the joint [47]. Numerous studies have confirmed the feasibility and effectiveness of gradient adhesives in enhancing joint performance [48,49,50,51,52].

The finite element method (FEM) is a mature and reliable technique for accurately predicting adhesive joint strength, particularly well-suited for designing joints with relatively complex graded adhesives. Methods such as continuum mechanics, fracture mechanics, damage mechanics, cohesive zone models (CZM), and extended FEM (XFEM) are commonly used for the adhesive failure analysis [53]. The failure modes of adhesive joints can generally be classified into interface failure, cohesive failure, mixed failure, and substrate failure. The damage mode is primarily influenced by the interface strength, adhesive strength, and the strength of the bonded substrate [54]. Kim et al. [55] employed a finite element model incorporating mixed-mode continuous damage to predict the failure and damage behavior of functionally graded bonded joints. They further analyzed the failure envelope within a design variable space, considering various material and geometric parameters. Nimje et al. [56] conducted a three-dimensional finite element analysis to investigate the damage behavior of functionally graded bonded double-lap joints (DLJs) with laminated FRP composites, identifying the critical locations where damage initiates. Using an elastic-plastic model and a mixed-mode cohesive damage model, Dadian et al. [57] analyzed stress distributions and predicted failures in functionally graded aluminum/GFRP adhesive lap-shear joints, increasing the shear load strength of graded specimen.

Given that SikaPower^®^-1511 and SikaPower^®^-1548 are two novel adhesives yet to be commercialized, a thorough understanding of their thermal curing behavior is crucial for designing graded adhesives. The performance of gradient adhesives largely depends on the compatibility of thermal and mechanical properties between the constituent adhesives. Therefore, this study systematically investigates the thermal curing behavior of SikaPower^®^-1511 and SikaPower^®^-1548 using DSC and FTIR, while experimentally determining their mechanical properties to ensure their effective integration in gradient adhesive configurations.

It is worth noting that SikaPower^®^-1511 and SikaPower^®^-1548 are two relatively similar epoxy resin materials, both regarded as tough adhesives, in contrast to the two highly dissimilar adhesives commonly utilized in current research on mixed joint design. This study emphasizes their distinct curing behaviors and differences in thermal and mechanical properties, offering a novel perspective on utilizing similar adhesives to achieve graded joint performance.

Beyond the individual evaluation of each adhesive’s bond strength, two graded joint models are analyzed in detail. Specifically, four adhesive configurations, including single adhesive and graded adhesive models are examined to reflect the influence of adhesive placement on joint performance and to meet diverse application requirements.

A high-precision finite element analysis was conducted to capture stress distributions near singularities in graded joints. Using fine meshing with a minimum element size of 0.1 μm, and controlling simulation errors within 1%, the analysis accurately depicted stress concentrations at the adhesive interface. This approach advances the understanding of failure mechanisms in graded adhesives under complex stress states, providing valuable references for their design. Unlike most existing studies, which primarily focus on overall stress distribution or the adhesive’s central layer, this study highlights the critical stress behavior near interface ends and singularities.

The objective of this study is to integrate experimental investigations with high-precision finite element analysis to compare the bond strengths of SikaPower^®^-1511 and SikaPower^®^-1548 in traditional single-adhesive designs and graded adhesive designs. It examines the effects of adhesive configuration on joint strength, stress concentration and potential failure mechanisms under tensile loading conditions. This work advances current understanding and provides a reliable theoretical foundation and technical guidance for optimizing the design and epoxy resins application of graded adhesives.

## 2. Materials and Methods

**Figure 1:**
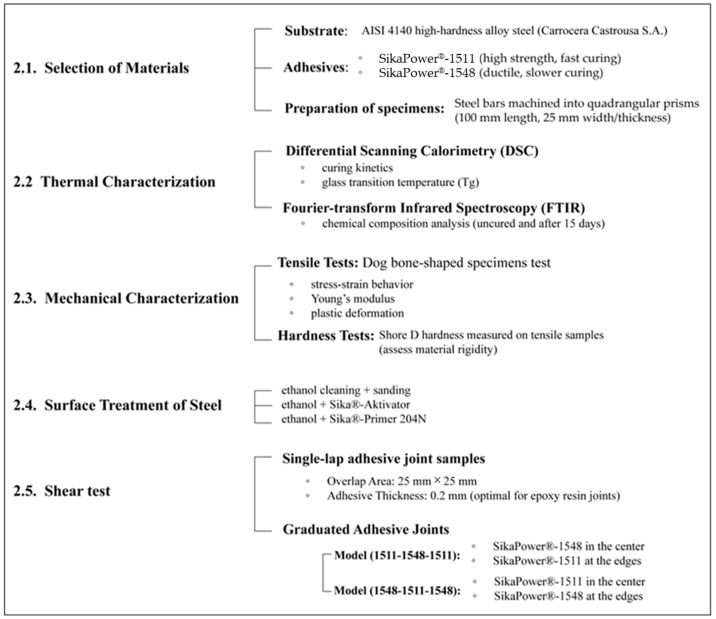
Diagram of Research Implementation.

The experimental workflow and procedures discussed in this chapter are summarized and visually presented in Figure 1, providing an overview of the research implementation.

### 2.1. Selection of Materials

The substrate selected for testing is a high-hardness alloy steel, AISI 4140, provided by Carrocera Castrousa S.A. (Santiago de Compostela, Spain). This steel possesses high rigidity, which minimizes deformation under tensile stresses, its chemical composition and mechanical properties can be found in Table 1 (provided by the supplier). To prevent buckling during testing, producing specimens with the correct thickness and length will be essential. Steel bars were machined using a Kondia B-500 CNC milling machine (Kondia M.E Taldeo S.L., Elgoibar, Guipúzcoa) into quadrangular prisms measuring 100 mm in length, with a base width and thickness of approximately 25 mm. This thickness was chosen based on a study that examined its effect on adhesion forces in steel under identical tensile load conditions, concluding that peeling stress decreases with increasing thickness and stabilizes around 25 mm [58]. Thus, peeling stress can be considered practically negligible, with the maximum observed stress attributed primarily to pure shear forces.

In the study of graded adhesive bonds, two structural epoxy resins were used: SikaPower^®^-1511 and SikaPower^®^-1548, supplied by Sika S.A.U. (Alcobendas, Spain). These adhesives belong to the same family and are both two-component systems mixed in a 1:1 ratio. These adhesives are versatile structural adhesives with no designated specific application. According to the technical data sheets, SikaPower^®^-1511 offers very high curing speed and high strength [59], whereas SikaPower^®^-1548 is more ductile with strong mechanical performance, albeit with a slower curing time [60]. Thermal and mechanical characterization provided further insight into the differences between these epoxy resins.

### 2.2. Thermal Characterization of Adhesives

Both adhesives were characterized in their uncured state to analyze their curing kinetics using differential scanning calorimetry (DSC) and Fourier-transform infrared spectroscopy (FTIR). DSC was also employed after curing to determine the glass transition temperature (T_g_).

#### 2.2.1. Differential Scanning Calorimetry

Thermal tests were conducted using a DSC822 device (Mettler Toledo, Greifensee, Switzerland). Adhesive samples, weighing between 8–10 mg were placed in small aluminum crucibles with a capacity of 40 µL. The reference sample was an empty crucible of the same size. Both crucibles were covered with lids, each with an approximately 50 µm hole. Sample temperatures were measured with high-precision thermocouples and controlled by an intercooler connected to the device, enabling temperatures as low as −60 °C.

To calculate the kinetics of the uncured epoxy resins, non-isothermal procedures were programmed from 0 to 200 °C at various heating rates (5, 10, 15, and 20 °C/min). The same non-isothermal method was applied to determine the glass transition temperature (T_g_) of the cured samples, with a heating rate of 10 °C/min.

The curves generated at different heating rates were analyzed using STARe software (Mettler Toledo, Greifensee, Switzerland), version 12.10, applying the MFK free kinetics model [61]. As all kinetic models are empirical, the degree of conversion (α) for the non-isothermal tests were calculated using the MFK model. After processing these values, activation energy (E_a_) was calculated as a function of α. Additionally, this approach allowed for evaluating kinetic reactions by simulating isothermal curves at various temperatures.

Previous studies have demonstrated the validity of this method for calculating E_a_ in materials similar to the epoxy resins used in this project [62].

#### 2.2.2. FTIR Spectroscopy

In this study, the curing process and composition of adhesives are examined through Fourier transform infrared spectrometry with attenuated total reflectance (FTIR-ATR) to obtain precise information about the chemical bonds and functional groups involved. The analysis focuses on the wavenumber range from 600 to 3600 cm^−1^, where the infrared absorption bands correspond to the specific vibrational frequencies of functional groups crucial to understanding the adhesive’s chemical structure.

The FTIR-ATR spectra are recorded using a Bruker Tensor 27 model (Bruker Optik GmbH, Ettlingen, Germany), employing the ATR technique to enhance spectral quality. This technique involves using a diamond ATR prism with an incident radiation angle of 45°, a configuration that allows effective penetration of the infrared beam into the sample.

Prior to each measurement, background spectra are recorded and subtracted to remove any interference from environmental humidity or CO_2_ present in the air, which could otherwise distort the results. Both components are analyzed separately, immediately after mixing, and again after 15 days.

### 2.3. Mechanical Characteristics of Adhesives

The mechanical properties of epoxy resins can be assessed in various ways. In this study, the characteristics most relevant to the design of graded joints were analyzed. Tensile and hardness tests were conducted to determine their resistance to stress, modulus of elasticity, and toughness. Understanding these properties is essential for effectively using these materials in future tests.

While the epoxy adhesive supplier provides approximate values for each product’s properties, it was necessary to quantify these values precisely. For this project, the resins were cured at room temperature for all tests, with a curing period of 15 days to ensure full curing, in a controlled environment at 22 °C and 35% relative humidity.

#### 2.3.1. Tensile Test

**Figure 2:**
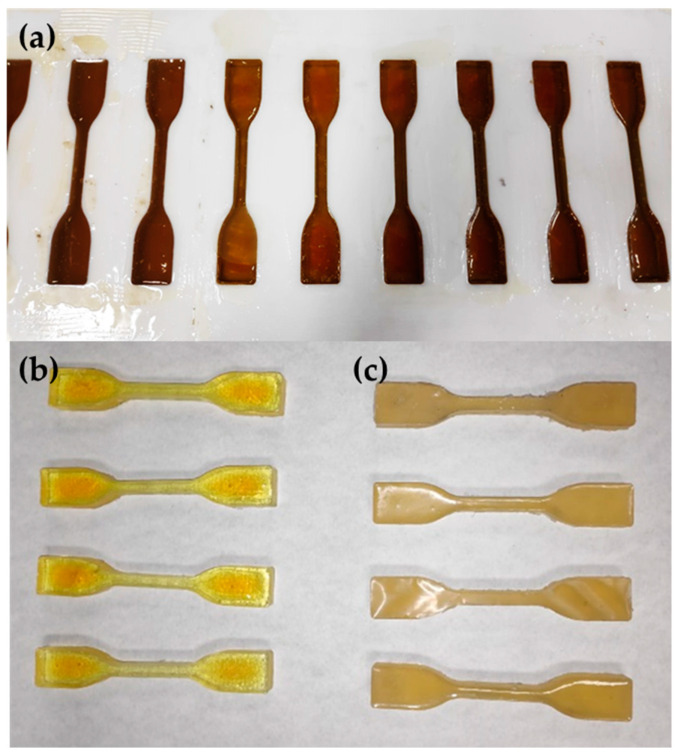
(**a**) Teflon mold with samples in manufacturing process, (**b**) samples of adhesive SikaPower^®^-1511 and (**c**) samples of adhesive SikaPower^®^-1548.

The tensile test specimens were manufactured by casting, pouring the epoxy resin mixture into a Teflon mold lined with Teflon tape at the bottom to prevent leaks. The mold was positioned between two Teflon sheets with a weight placed on top for stability. The specimens were shaped as dog bones, with a central section measuring 5 ± 0.5 mm thick, 10 ± 0.5 mm wide, and 115 mm in length. Five samples were tested for each adhesive, although ten samples of each epoxy resin were manufactured. Figure 2a shows the samples in the mold and Figure 2b,c correspond to samples of adhesive SikaPower^®^-1511 and SikaPower^®^-1548 respectively.

The tensile tests were conducted in accordance with UNE-EN ISO 527-1 [63], using a universal testing machine provided by Microtest (Madrid, Spain). A 5 kN load cell was used for the SikaPower^®^-1511 adhesive and a 20 kN load cell for the SikaPower-1548 adhesive, with a test speed of 2 mm/min.

The tensile strength-strain data were calculated from the force-displacement curves. To measure plastic deformation, marks were made on the narrow section (gauge length) of each sample, establishing an initial gauge length (L_0_) of 37 mm. The stress-strain curve enabled the determination of the elastic limit, Young’s modulus, and both elastic and plastic deformation for each adhesive—values essential for simulating adhesive joints made with these materials.

#### 2.3.2. Hardness Test

Shore D hardness was measured according to ASTM D2240-15 [64]. Shore D is used for hard elastomers, plastics, and rigid thermoplastics. The indenter has a cone angle of 30°, and the spring force is 44.5 N. A Bareiss durometer (Oberdischingen, Germany) was used for the measurements. The test was performed 10 times on the heads of the tensile samples, repeating this process across all 5 samples. Therefore, the hardness value of each adhesive is an average of 50 measurements.

### 2.4. Surface Treatment of Steel

Surface treatments are essential to ensure good adhesion between the substrate and the adhesive, with the goal of achieving cohesive failure within the adhesive bond. In this study, three different treatments were compared: (1) cleaning with ethanol, sanding with 180-grit sandpaper, and a final cleaning with ethanol; (2) cleaning with ethanol followed by the application of Sika^®^-Aktivator (Sika, S.A.U., Alcobendas, Spain); and (3) cleaning with ethanol and application of Sika^®^-Primer 204 N (Sika, S.A.U., Alcobendas, Spain).

To select the optimal treatment, three simple shear overlap samples were prepared per treatment using SikaPower^®^-1548 adhesive, which is the most ductile and has a slower curing time according to its technical data sheet—potentially causing more curing challenges.

### 2.5. Shear Tests

The shear test was conducted on single-lap adhesive joint samples. These samples were created from pre-cut, surface-treated steel squares. The overlap area was 25 × 25 mm to prevent peel stress. The adhesive was applied on both surfaces with a thickness of 0.2 mm, a value shown by Silva et al. [65] to be optimal for epoxy resin joints. This thickness was achieved by placing metal shims of the same thickness on the steel pieces.

**Figure 3:**
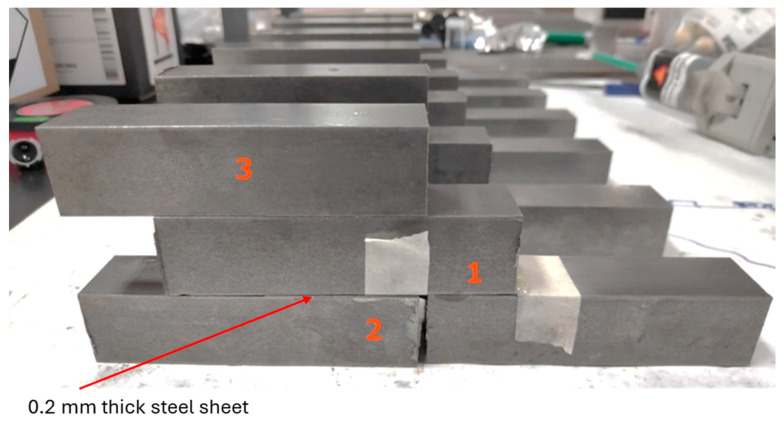
Assembly of square steel prisms for adhesive bonding. Area (1): adhesive zone. Prism (2): support. Red arrow: placement of steel shim to achieve 0.2 mm thickness. Prism (3): stabilizes the assembly.

Figure 3 illustrates the adhesive application area (1). Masking tape was placed along the edges of each block’s adhesive area to facilitate removal of any excess adhesive that might overflow. Additionally, a support object (in this case, a sample of the same steel) was placed beneath the steel block with adhesive on its underside (2). The arrow indicates the location where the steel sheet with a 0.2 mm adhesive layer was applied for optimal bonding. To prevent the resin from adhering to the support block, it was coated with wax in the area closest to the bond. Furthermore, to keep the steel blocks stable during the curing process and to maintain their spacing, an additional steel prism was placed on top (3), with careful attention to avoid exerting extra pressure on the adhesive area.

The primary aim of this study is to design graduated adhesive joints. In addition to evaluating each adhesive individually, two graduated joint models were analyzed for comparison, resulting in a total of four different single-lap adhesive joint models.

As mentioned in the project introduction, the maximum stress in this test typically occurs at the edges of the adhesive-adherent interface, specifically at one of the corners. To address this, the graduated joints were designed so that one of the adhesives is applied in the central area (15 mm), while the other is used only at the edges (5 mm on each side), achieving a total overlap of 25 mm. Due to the substantial thickness of the test specimens, peel stress is minimized, allowing the assumption that maximum stress will be limited to shear stress alone.

In model (1511-1548-1511), SikaPower^®^-1548 (more ductile) was applied in the center of the joint, with SikaPower^®^-1511 (stronger) at the edges. For the last model tested, specimens were fabricated in the reverse configuration: the rigid adhesive (SikaPower^®^-1511) was used in the central area, with the ductile adhesive (SikaPower^®^-1548) applied at the edges, creating model (1548-1511-1548).

## 3. Results

### 3.1. Curing Kinetic of the Selected Adhesives

**Figure 4:**
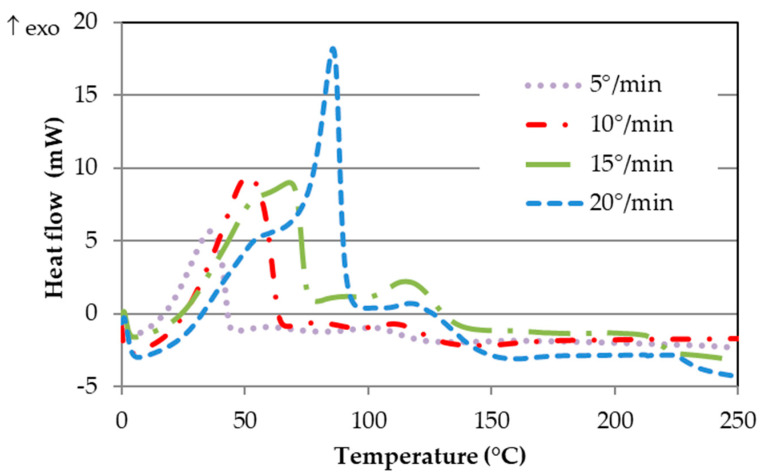
Curing curves at different rates for SikaPower^®^-1511.

Figure 4 shows the curves at different rates for SikaPower^®^-1511. Similar plots were found for SikaPower^®^-1548 but a single peak. It is worth noting the presence of two curing peaks in this adhesive at heating rates of 20 °C/min and 15 °C/min, which are less pronounced at 10 °C/min and 5 °C/min. Higher DSC heating rates lead to more distinct peaks and a shift of these peaks to higher temperatures within shorter time frames. The two curing peaks, discussed in Section 5, may be attributed to the combination of different compounds in the hardener.

The maximum curing peak temperatures and the total curing enthalpies for both adhesives are shown in Table 2. To ensure data reliability, three thermograms were conducted for each heating rate and adhesive. The values in Table 2 represent the average of these measurements, and the associated errors are calculated as the standard deviation. It can be observed that the first curing peak for SikaPower^®^-1511 occurs at a relatively low temperature, while the second peak is above 100 °C, similar to the curing peak temperature of SikaPower^®^-1548 adhesive. Regarding the curing enthalpy, SikaPower^®^-1548 releases more heat during the process, approximately 100 J/g more than SikaPower^®^-1511. Specifically, the enthalpy for SikaPower^®^-1511 is 203 ± 1 J/g, whereas for SikaPower^®^-1548, it is 298 ± 2 J/g, making it a more exothermic reaction.

**Figure 5:**
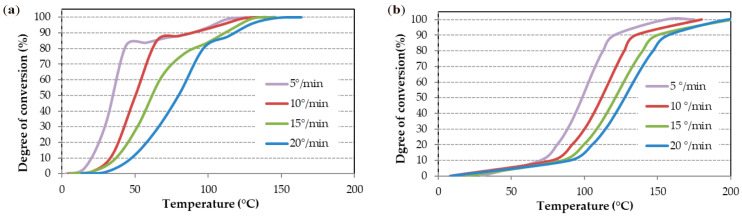
Degree of conversion curves at different rates for (**a**) SikaPower^®^-1511 and (**b**) SikaPower^®^-1548.

The degree of conversion versus temperature for the four heating rates are shown in Figure 5. In the case of the SikaPower^®^-1511 adhesive (Figure 5a), a noticeable change in slope between 85% and 90% conversion is observed at heating rates of 20 °C/min and 15 °C/min, consistent with the two curing peaks seen in Figure 3. For this adhesive, conversion begins below 50 °C and proceeds rapidly up to 80–85%, after which it slows down. However, for SikaPower^®^-1548 (Figure 5b), conversion starts more slowly, accelerating significantly around 100 °C at roughly 10% conversion. This rapid curing continues up to 90%, where the slope changes again and the reaction rate slows, ultimately reaching full conversion at nearly 200 °C. These observations indicate that the curing behavior is markedly different between the two adhesives.

**Figure 6:**
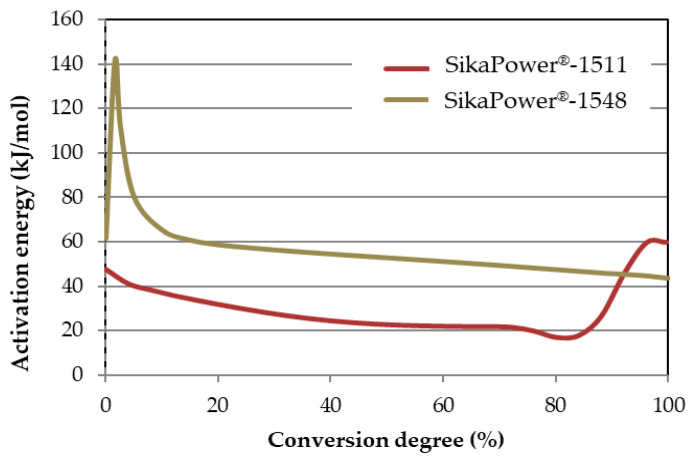
Activation energy curves by MFK for SikaPower^®^-1511 and SikaPower^®^-1548.

From the degree of conversion curves, the software calculates the E_a_ based on the degree of conversion, as shown in Figure 6 (see calculation details in Section 2.2.1). The SikaPower^®^-1548 adhesive exhibits a higher E_a_ throughout the process, particularly at the start of the reaction when the oxirane groups are being opened, with a difference of nearly 100 kJ/mol compared to SikaPower^®^-1511. In the middle stage of the reaction, while SikaPower^®^-1548 still has a higher E_a_, the difference narrows to about 30 kJ/mol. Starting at 80% conversion, the E_a_ of SikaPower^®^-1511 begins to increase, requiring more E_a_ to complete the reaction and reaching 99% conversion with an E_a_ that exceeds that of SikaPower^®^-1548 by 16 kJ/mol.

The simulation of isothermal curing at various temperatures (Table 3) shows curing times at 25 °C of approximately 12 to 12.5 h. As temperature increases, curing time decreases significantly, with curing completed in less than 10 min for SikaPower^®^-1511 and 20 min for SikaPower^®^-1548 at 100 °C. However, such rapid curing can lead to vitrification and is generally avoided. This simulation aligns with the observation that SikaPower^®^-1511 cures much faster up to approximately 90%, while curing for SikaPower-1548 is more gradual. This trend is also evident in the degree of conversion curves (Figure 5) and the lower temperature of the first curing peak (Table 2 and Figure 4).

**Figure 7:**
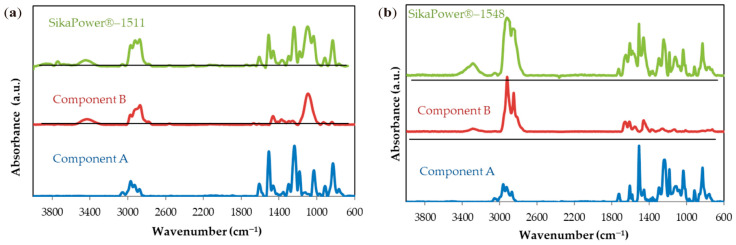
FTIR spectra for component A, component B and mixture for (**a**) SikaPower^®^-1511 and (**b**) SikaPower^®^-1548.

The infrared spectra of the two adhesives are shown in Figure 7. Since two-component adhesives are used, the resin (Component A), the hardener (Component B), and the mixture of both (M) are analyzed before the curing process.

The spectra of the resins in the two adhesives before curing are practically identical. The characteristic oxirane ring groups, located at 914 cm^−1^, will determine the curing index of the final resin.

The region between 720 and 830 cm^−1^ out of plane deformation and bending vibration corresponds to C-H bonds exhibiting out-of-s, present in both Components A and B of the two adhesives (Table 4 and Figure 7a,b). Around 914 cm^−1^, the oxirane ring exhibits a stretching vibration in both resins (Figure 7a,b). The decrease in this peak relative to the 1030 cm^−1^ peak over time allows monitoring of the resin’s degree of conversion. The peak at 1030 cm^−1^ corresponds to the C-O-C stretching vibration in aromatic rings, observed in both resins.

In the SikaPower^®^-1511 hardener—component B (Figure 7a), the highest intensity peak is seen at 1080 cm^−1^, likely associated with cyclic ethers. Additionally, primary amine C-N stretching vibrations are evident between 1020 and 1090 cm^−1^ for SikaPower^®^-1548 hardener (Figure 7b and Table 4).

In the 1100–1182 cm^−1^ range, more C-H (Table 4 and Figure 7a,b for component A) or phenol group deformations (Figure 7a component B) and bending vibrations are present, along with aromatic ring vibrations of the =C-O-C= bond. In the subsequent region at 1245–1290 cm^−1^, stretching vibrations of C-O-C (Table 4 and Figure 7a,b) and C-N bonds can be attributed to aromatic and unsaturated amines in hardeners (Figure 7a,b—components B).

For Component B of SikaPower^®^-1511 (Figure 7b), a peak at 1367 cm^−1^ likely corresponds to the CH-S deformation vibration of mercaptan (Table 4). Other weak bands associated with C-S stretching vibrations might appear around 710 cm^−1^ and 2600–2540 cm^−1^ but were not observed.

C-O bond stretching vibrations for both adhesives appear at 1450 cm^−1^ and may overlap with tertiary amine vibrations at 1456 cm^−1^ (Figure 7a,b). Aromatic ring stretching vibrations for C-C and C=C bonds are observed at 1508 cm^−1^ and 1607 cm^−1^, respectively, and for C=C bonds in phenols at 1650 cm^−1^. A strong absorption band at 1730 cm^−1^, due to the C=O stretching vibration in esters, is present in both adhesives.

Stretching bands of the =CH rings are observed at 3057 cm^−1^, overlapping with C-H stretching bands of methyl groups and terminal methanols. Symmetrical and asymmetrical interactions of methyl and methylene groups appear at 2926 cm^−1^ and 2966 cm^−1^ (Figure 7a,b and Table 4).

Beyond 3000 cm^−1^, peaks in the hardeners correspond to amines, with N-H stretching vibrations observed between 3360 cm^−1^ (notably in SikaPower^®^-1548) and 3416 cm^−1^ (SikaPower^®^-1511). This region also includes OH group stretching vibrations, absent in the adhesives’ spectra, suggesting a higher presence of N-H bonds in the hardeners.

In the spectra of the uncured mixtures, the characteristic groups of Component A and Component B appear combined without alterations in their respective components. Table 4 lists the most characteristic bands, and the vibration types associated with each functional group.

**Figure 8:**
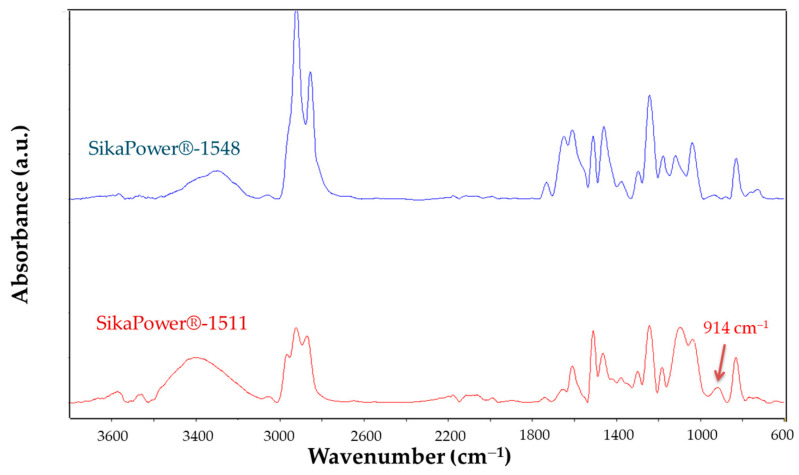
FTIR spectra of SikaPower^®^-1548 and SikaPower^®^-1511 after 72 h of curing.

The IR spectra of the resins cured for 72 h indicates whether the adhesive has fully cured (Figure 8). For SikaPower^®^-1548, no peak is observed at the band corresponding to oxirane rings (914 cm^−1^), confirming complete curing. However, for SikaPower^®^-1511, a small peak remains at 914 cm^−1^, indicating that curing is incomplete due to the presence of residual oxirane rings. This resin likely requires post-curing to complete the process. Although the adhesive’s strength may be adequate, extending the curing process can help ensure sufficient ductility.

### 3.2. Glass Transaction Temperature

The T_g_ indicates the degree of cross-linking in epoxy resin chains during the curing process, the T_g_ can be observed in Table 5. For SikaPower^®^-1511, the first scan shows an initial T_g1_ at 38 °C, followed by a second T_g2_ at 94 °C and a curing peak with an energy release of 10.39 J/g (5% of total). By the second scan, the resin is fully cured, showing T_g_ at 37 °C and 140 °C, indicating complete curing of the adhesive. However, for SikaPower^®^-1548, the first scan reveals an initial T_g1_ at 72 °C and a second T_g2_ at 129 °C, indicating complete curing in the first scan. With a significantly higher initial glass transition temperature than the SikaPower^®^-1511, SikaPower^®^-1548 exhibits greater chain cross-linking upon reaching this temperature.

### 3.3. Mechanical Properties of the Selected Adhesives

**Figure 9:**
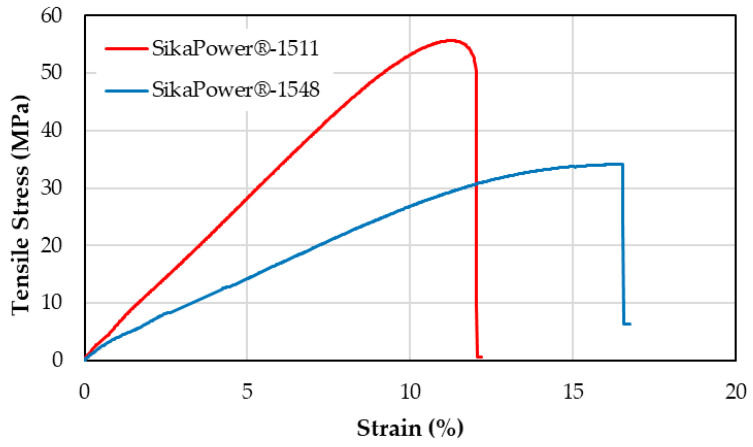
Representative tensile stress curves for SikaPower^®^-1548 and SikaPower^®^-1511.

Figure 9 presents the stress-strain curves for each adhesive, highlighting distinct differences: SikaPower^®^-1511 exhibits the highest tensile strength, whereas SikaPower^®^-1548 is the most ductile of the two resins. Table 6 provides the average values derived from 10 stress-strain curves from tensile tests, as well as the Shore D hardness for each epoxy adhesive. SikaPower^®^-1511 demonstrates a tensile stress that is 36% higher than that of SikaPower^®^-1548, while its strain is 45% lower, indicating a much stiffer but less deformable behavior. Additionally, SikaPower^®^-1511 has a Shore D hardness that is 10% greater than that of SikaPower^®^-1548, reinforcing its characterization as the stronger, though less ductile, adhesive. These characteristics are further supported by a Young’s Modulus for SikaPower^®^-1511 that is 45% higher.

### 3.4. Shear Behavior and Graduated Joints

Preliminary tests to select the optimal surface treatment were conducted using the SikaPower^®^-1548 adhesive. The best result in terms of deformation was achieved with Sika^®^-Aktivator, but cleaning with solvent alone produced similar deformation values and slightly higher strength. Consequently, simple solvent cleaning was chosen as the surface treatment, as all fractures exhibited adhesive failure.

**Figure 10:**
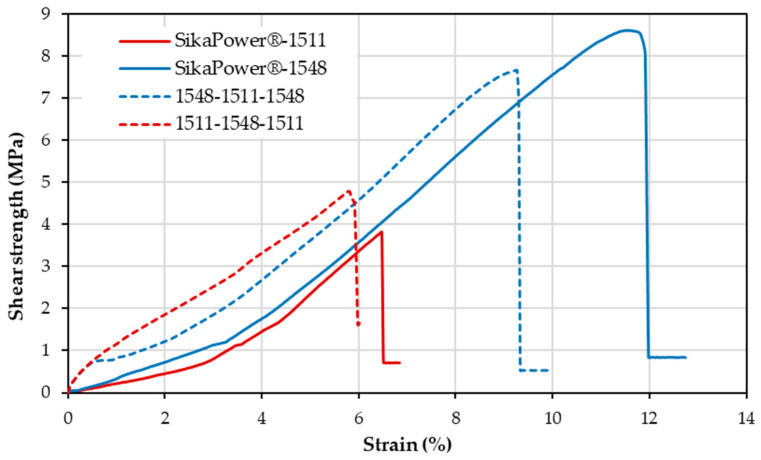
Representative shear strength curves for SikaPower^®^-1548, SikaPower^®^-1511 and graduated joints, with SikaPower^®^-1548 on the edges (1548-1511-1548) and SikaPower^®^-1511 on the edges (1511-1548-1511).

Initially, it was assumed that the resin with the highest Young’s modulus and tensile stress would also perform better in shear tests. However, the opposite was observed: the more ductile and less rigid resin outperformed in terms of maximum shear strength and strain (Table 7 and Figure 10). The simple, single-adhesive bond with SikaPower^®^-1548 (the more ductile resin) withstood shear stresses more than twice as high as those with SikaPower^®^-1511 (9 MPa versus 4 MPa). The greater strain capacity of SikaPower^®^-1548, resulting from its higher ductility, is key to withstanding shear stresses more effectively. This flexibility allows it to absorb and distribute the load better, whereas rigid bonds, like those with SikaPower^®^-1511, are more prone to failure under concentrated shear stress.

Shear tests indicate that graded adhesive joints can enhance the strength of bonded assemblies. Combining a more ductile resin with a stiffer resin increases the maximum shear stresses these joints can withstand compared to using only SikaPower^®^-1511 (rigid). For instance, when SikaPower^®^-1548 is applied at the ends and SikaPower^®^-1511 in the center (1548-1511-1548), the maximum shear stress doubles, reaching 8 MPa compared to 4 MPa with SikaPower^®^-1511 alone (Table 7 and Figure 10). Reversing the configuration, with SikaPower^®^-1511 at the ends and SikaPower^®^-1548 in the center (1511-1548-1511), also yields a modest increase in strength, reaching 5 MPa versus 4 MPa with SikaPower^®^-1511 alone (Table 7 and Figure 10). Thus, the performance of a joint using an adhesive with a high Young’s modulus can be improved by incorporating an adhesive with a lower Young’s modulus, either at the ends or in the center, specifically under shear stresses. The more ductile adhesive, SikaPower^®^-1548, still demonstrates superior deformation performance in shear tests compared to the graded joint. However, the strength of SikaPower^®^-1548 alone is only slightly higher than that of the graded joint configuration (1548-1511-1548).

Since both adhesives exhibit similar shear behavior and the differences in strength and strain are small, it can be said that the graduated joints maintain the closed strength as the adhesive at the edges. For example, the graduated joint 1548-1511-1548 has a strength of 8 MPa, compared to 9 MPa for the 1548 adhesive. In terms of strain, it decreases slightly from 11% for the 1548 adhesive to 9% for the graduated joint. The other graduated joint, 1511-1548-1511, has a strength of 5 MPa, matching the 4 MPa strength of the 1511 adhesive. In this case, the strain remains unchanged. The use of more different adhesives would make these differences more noticeable.

It should be noted that the shear strength and strain values represent averages from five specimens, with errors indicated by the standard deviations of the measurements. The relatively high measurement error is primarily attributed to the manual preparation of the samples, where small variations in adhesive thickness can significantly influence the shear strength. Specifically, for SikaPower^®^-1511, the error fundamentally arises from differences in the degree of curing among samples, as minor variations in cross-linking result in substantial changes in the material properties.

## 4. Numerical Analysis

The Finite Element Method (FEM) is a powerful numerical simulation tool widely used in joint strength analysis and structural design due to its ability to accurately predict complex stress distributions and failure mechanisms in graduated bonded joints. In this study, finite element models of single-lap adhesive joints using SikaPower^®^-1511 and SikaPower^®^-1548 two epoxy resins are developed in the commercial software Abaqus CAE (https://www.3ds.com/products/simulia/abaqus).

### 4.1. Model Development

#### 4.1.1. Material Properties

The adherend material used in the finite element simulation is alloy steel AISI 4140, which is the same as that used in the experiments, and the adherends Steel model is approximated as an elastic solid. The material properties are in accordance with Table 1 and the following mechanical properties are set: Young’s modulus E of 215 GPa, Poisson’s ratio of 0.3, and tensile strength of 1020 MPa. Due to the distinct plastic behavior of the resin materials, The SikaPower^®^-1511 and SikaPower^®^-1548 two epoxy resins are modeled as elastic-plastic solids with the experimental Stress-Strain curve. The material property parameters of the two epoxy resins are set as shown in Table 8.

#### 4.1.2. Joint Geometries and Conditions

**Figure 11:**
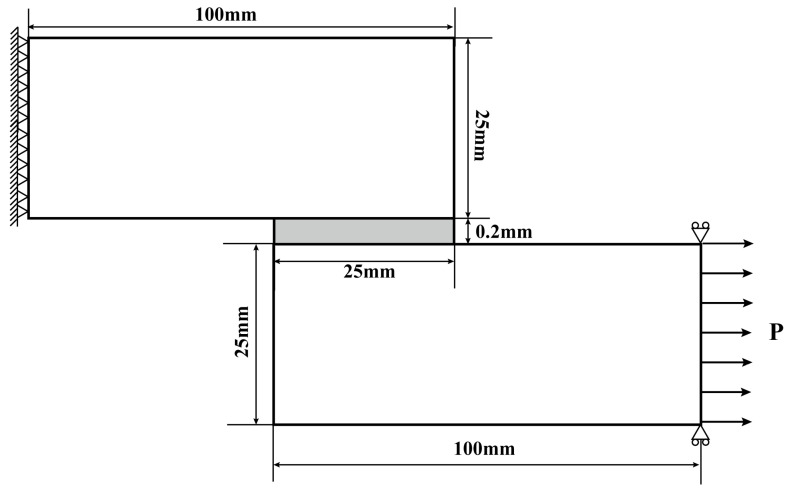
Geometry, dimensions, boundary, and loading conditions of the single lap joint. The adhesive is located in the gray area.

The single lap joint under horizontal tensile load is treated as a two-dimensional model of plane stress solid element. The geometry of the single lap joint model is consistent with the experimental specimen, with the steel adherends set to dimensions of 100 mm in length, 25 mm in width, and 25 mm in thickness. In this bonding model, the adhesive layer is configured as a rectangular section with dimensions of 25 mm by 25 mm and a thickness of 0.2 mm. The comprehensive geometry, dimensional specifications, boundary constraints, and loading conditions applied to the model are illustrated in Figure 11.

For the finite element static simulation analysis of the lap shear test, fixed constraints are applied to the left end of the upper adherend, while loads parallel to the lap region are imposed to the right end of the lower adherend.

#### 4.1.3. Meshing

**Figure 12:**
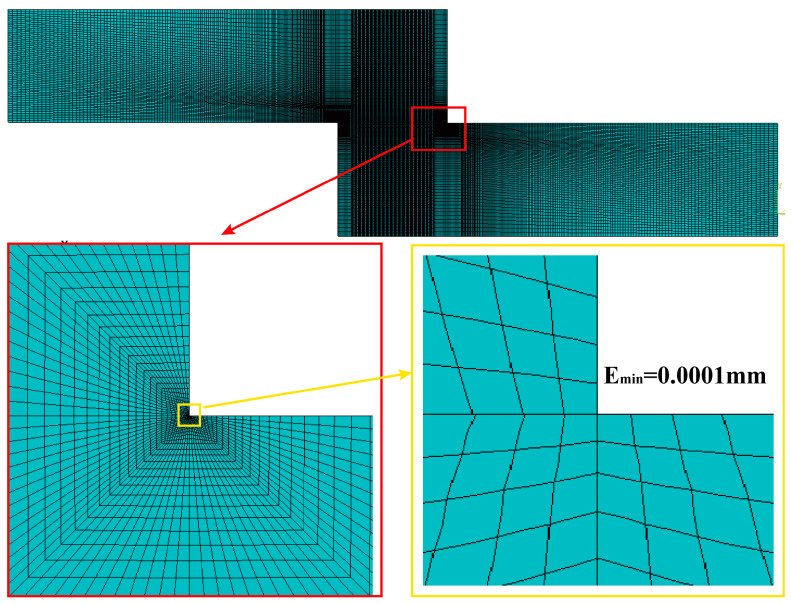
Local mesh refinement of the bonding interface in the lap joint.

When an adhesive structure is subjected to horizontal tensile loading, a region with a high-stress gradient forms at the end of the adhesive layer. The 2D plane stress assumption is adopted for thin-layer structures subjected to shear loads. Eight-node elements, utilizing quadratic interpolation functions, are employed to more accurately capture stress and strain distributions in regions of stress concentration compared to four-node (linear) elements. Full integration is applied instead of reduced integration to minimize numerical errors. To enhance calculation accuracy, the local mesh refinement method is applied in the stress concentration region, particularly around the stress singularity location. The mesh size near the end of adherend and adhesive bonding interfaces is controlled to a minimum of 0.1 µm, while in the non-refined regions, the maximum mesh size is 0.1 mm. The local mesh refinement is illustrated in Figure 12.

After meshing, a corresponding sensitivity analysis of the results was conducted. Models with minimum element sizes of 0.1 μm, 0.2 μm, 0.5 μm, and 1 μm were employed to analyze the peel stress distribution at each node. Further details are provided in Section 4.3.1.

Damage to the adhesive layer frequently occurs at points of stress singularity. When the mesh size accuracy reaches a minimum of E_min_ = 0.1 μm, finite element simulation results can more precisely characterize the singular stress distribution at the interface edge. This increased accuracy also enhances the precision of failure analysis for the epoxy resin adhesive.

### 4.2. Simulation Process

#### 4.2.1. Functionally Graded Adhesive Joints

**Figure 13:**
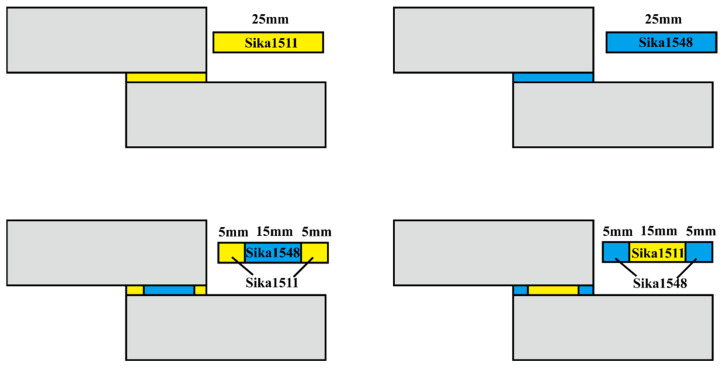
Traditional single-lap joints and graduated adhesive joints.

Different single-lap adhesive bonding simulated models, including traditional and graduated joints, were created by altering the configuration of the epoxy resin, based on the experimental setup of four joint models. The four models (schematically shown in Figure 13) were identical in overall dimensions, each featuring a bond length of 25 mm, but differed in the configuration of the epoxy resin.

#### 4.2.2. Simulation Analysis

The failure mode of bonded joints is influenced by several factors, including joint geometry, adhesive material properties, and the applied load. Key considerations such as the adhesive’s ductility and fracture toughness play a critical role, as they directly impact the location of crack initiation and its propagation, subsequently affecting the joint’s failure mode and overall structural strength. For accurate strength predictions in adhesive joints, selecting material damage and stiffness degradation criteria aligned with the adhesive’s ductility properties is crucial.

The maximum principal stress criterion is appropriate in applications using rigid adhesives like SikaPower^®^-1511, which exhibit high strength but low ductility. This criterion effectively predicts failure in materials subjected to high stress under minimal deformation. Conversely, for ductile adhesives such as SikaPower^®^-1548, which can undergo significant plastic deformation under load, the ductile damage criterion is more suitable, allowing for a more accurate representation of the material’s resistance to load through plastic deformation. This distinction ensures that predictions reflect the adhesive’s specific response to applied forces, leading to more reliable structural performance assessments.

### 4.3. Numerical Results

#### 4.3.1. Mesh Sensitivity Analysis

A mesh sensitivity analysis was conducted to verify the convergence of the numerical model established in the previous section and evaluate the influence of mesh size on computational accuracy. A local mesh refinement method was applied, and finite element analyses were performed on four models using SikaPower^®^-1511 adhesive with minimum mesh sizes of 0.1 μm, 0.2 μm, 0.5 μm, and 1 μm, respectively. These analyses were carried out under a horizontal load of P = 2 MPa.

The elements near the singular stress points at the adhesive interface ends were selected for detailed evaluation, and the peel stress at each node was used as a representative indicator to assess the impact of the mesh size.

**Figure 14:**
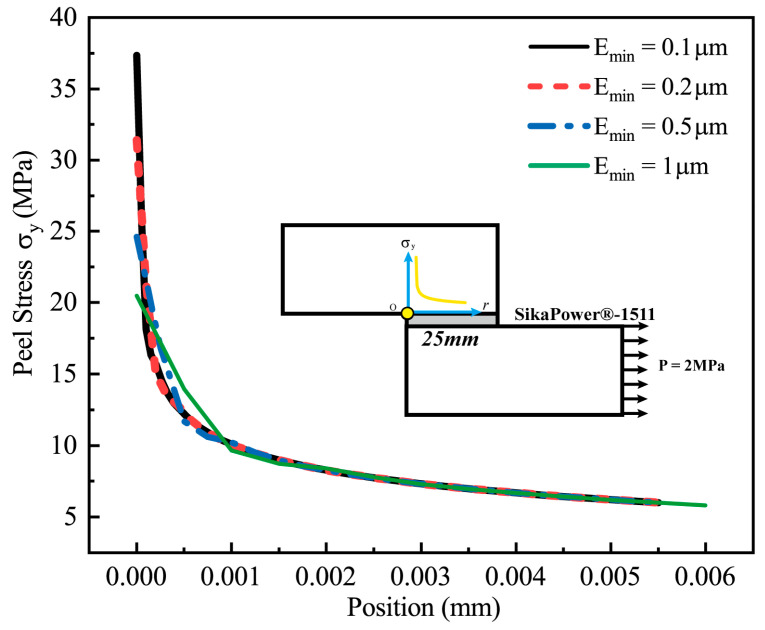
Convergence analysis of peel stress (σ_y_) near the adhesive interface endpoint using SikaPower^®^-1511 adhesive under Pressure = 2 MPa.

Figure 14 illustrates the distribution of peel stress near the singular stress point at the end of the adhesive interface for different mesh sizes.

Table 9 provides the peel stress values at the nodes of each interface element at varying distances from the singular point. Additionally, the table includes the ratio σ_0.2_/σ_0.1_, representing the peel stress values obtained with 0.2 μm and 0.1 μm mesh sizes, to quantify the calculation accuracy.

The results indicate that, in regions distant from the singularity, the peel stress remains largely independent of the mesh size. For instance, at r = 0.05 mm, the calculated peel stress values from all four models are nearly identical. However, closer to the stress singularity, the sensitivity of the peel stress to the mesh size increases. At r = 0 (the singularity point), the peel stress value is a singular result and cannot be accurately determined due to the inherent limitations of finite element calculations. As mesh resolution increases, the edge nodes at r = 0 yield progressively higher numerical stress values, with σ_0.2_/σ_0.1_ = 0.841.

Excluding the singularity at r = 0, the peel stress stabilizes quickly as the distance increases. For example, at the second node, located at r = 0.0005 mm, the peel stress ratio is already close to unity (12.343 MPa/12.214 MPa ≈ 1.0106).

The results demonstrate that when the minimum mesh size is reduced to 0.2 μm, the peel stress values near the adhesive interface begin to converge. Further refinement to 0.1 μm reveals that, except at singular points, the relative error at other nodes is less than 1%. This refinement provides a more accurate representation of the stress distribution in regions with high stress gradients.

Considering the controllable computational cost, a finer mesh size of 0.1 μm was adopted in this study for local refinement to effectively capture the characteristics of stress concentration.

#### 4.3.2. Experimental and Numerical Failure Strength

Using the finite element simulation model developed, the numerical shear lap strengths of conventional joints and gradient joints, with SikaPower^®^-1511 and SikaPower^®^-1548 epoxy resins as adhesives, were calculated. A preliminary comparative analysis of these results with experimental findings is presented in Table 10. (Shear strength at failure was determined by dividing the failure load by the bonding area).

The numerically simulated and experimental results obtained from the four models, encompassing both conventional and gradient joints, exhibit good overall consistency.

For the conventional joints using SikaPower^®^-1511 and SikaPower^®^-1548 as single adhesives, the shear strengths predicted by numerical simulations were 4.515 MPa and 8.274 MPa, respectively, showing good agreement with the experimental results of 4 ± 2 MPa and 9 ± 3 MPa. This consistency indicates that the finite element model developed in this study effectively predicts the shear strength of traditional single-adhesive joints.

However, for the gradient joints with different adhesive configurations, some discrepancies were observed between the simulation and experimental results. For instance, with the 1511-1548-1511 adhesive configuration, the simulated shear strength was 4.115 MPa, compared to an experimental strength of 5 ± 1 MPa; whereas, with the 1548-1511-1548 configuration, the simulated shear strength was 10.941 MPa, while the experimental result was 8 ± 2 MPa, showing some differences.

The discrepancies between the numerical and experimental results may stem from the assumption in the numerical simulation that the adhesive materials are ideal, uniform, and isotropic, which does not fully account for the microstructural characteristics of the adhesive interface or variations in material properties. The two different epoxy resins SikaPower^®^-1511 and SikaPower^®^-1548 exhibit different thermal curing behaviors under identical heating conditions, resulting in a more complex curing process in combined adhesive joints. In actual experimental conditions, it is crucial to consider the impact of the epoxy resin’s degree of curing on the shear strength of the adhesive joint during the thermal curing process.

Furthermore, in a gradient joint combining two epoxy resins, interfacial penetration, chemical bonding, or other interfacial reactions may occur between the two adhesive layers. Any fluctuation in interfacial bond strength during experiments could directly affect the overall shear strength of the combined joint, leading to variations in the experimental results. The influence of the thermal curing process on the material properties of the two combined epoxy resins may be a key source of discrepancy between the experimental and numerical simulation results. Nonetheless, the numerical simulations and experimental results exhibit a consistent trend, with a certain degree of error, but within a reasonable range. This consistency validates the reliability of the established finite element model and highlights the impact of epoxy adhesive on the shear strength of both conventional and gradient joints.

#### 4.3.3. Stress Analysis

Analyzing stress distribution in critical regions of the lap structure is essential for accurately predicting the failure mode and ultimate strength of the joint. In this section, the peel and shear stress values at each nodal point on the adhesive interface and within the adhesive layer were extracted from the finite element analysis under various loading conditions. Stress distribution was visualized by plotting position-stress curves along the horizontal direction of the adhesive joint, starting from the bonding edge.

The interface, defined as the contact surface between two different materials, plays a crucial role in joint integrity [67]. When the adhesive and adherend form an interface with low adhesion—often due to unsuitable material selection or poor surface quality—interfacial peeling failure of the adhesive layer and adherend is likely to occur. Consequently, avoiding interfacial failure is a key consideration in joint design. When the joint interface strength is high, cohesive failure may occur because of the adhesive’s limited intrinsic strength.

In adhesive joints, the stress distribution within the adhesive layer significantly affects the overall joint performance.

**Figure 15:**
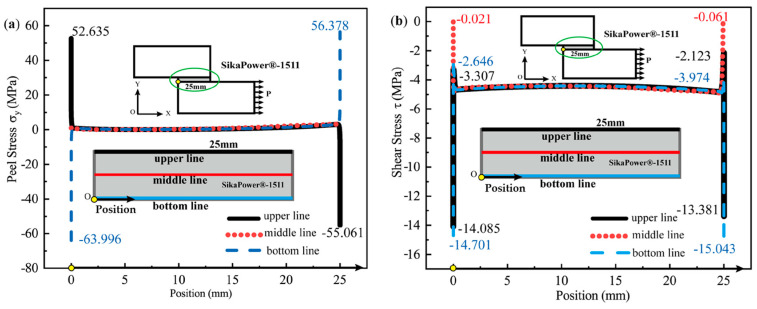
Stress distribution at the adhesive interface and within the adhesive layer of SikaPower^®^-1511. (**a**) Peel stress distribution; (**b**) Shear stress distribution. The gray area indicates the location of the adhesive, it is marked with a green eclipse.

Three sections are selected for stress distribution analysis: the upper interface (where the adhesive bonds to the adherends), the middle of the adhesive layer, and the lower interface of the adhesive bond. Each section is aligned along the horizontal load application direction, corresponding to the bond line of the adhesive layer. As illustrated in the simplified bonding diagram in Figure 15, the upper interface section is represented by a black line, the middle adhesive layer section by a red line, and the lower interface section by a blue line. In finite element simulation analysis, axis orientation can influence the sign convention for stress direction. In this study, the model’s axes are set as follows: the x(1) axis points horizontally to the right, and the y(2) axis points vertically upward. Therefore, peeling stress $\sigma_{y}$($S_{22}$) along the y-direction and in-plane shear stress $\tau_{xy}$($S_{12}$) along the x-direction in the x–y plane are extracted. Additionally, for the epoxy adhesive layer with a length of 25 mm, to analyze the stress distribution along the horizontal section line, the left end point of the section line is designated as the origin, and the distance from the origin to the adhesive element node position is used as the x-coordinate to extract the node stress value.

Taking SikaPower^®^-1511 as an example, the distribution patterns of peel and shear stress at the adhesive interface and within the adhesive layer under critical failure load conditions are presented in Figure 15a and Figure 15b, respectively.

When the thickness of the bonded parts reaches 25 mm or more, the peel stress in the adhesive layer resulting from the joint’s bending deformation is significantly reduced. In the peel stress distribution in Figure 15a, the peel stress across the middle section of the adhesive layer approaches zero and is nearly negligible.

The peel stress distribution curves at the upper and lower interfaces of the adhesive layer are symmetrical about the centerline, with pronounced stress singularities at the interface ends where the peel stress is significantly elevated. The maximum positive peel stresses occur at the left end of the upper interface ($\sigma_{y}\;$= 52.635 MPa) and at the right end of the bottom interface ($\sigma_{y}\;$= 55.052 MPa). The maximum negative peel stresses are observed at the right end of the upper interface ($\sigma_{y}\;$= −55.061 MPa) and the left end of the lower interface ($\sigma_{y}\;$= −63.995 MPa). In the central region of the adhesive layer, away from the endpoints, the peel stress remains at a small, nearly zero value.

**Figure 16:**
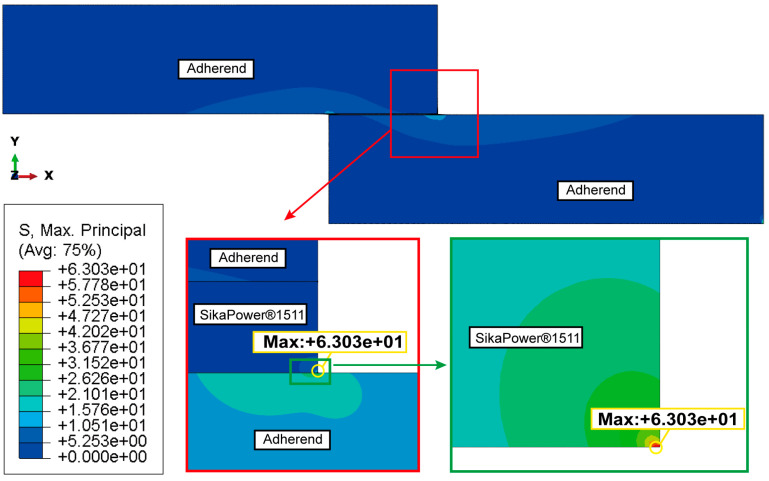
The maximum principal stress distribution of SikaPower^®^-1511 adhesive.

Figure 15b illustrates the distribution of shear stress $\tau_{xy}$ along both the interface and the centerline of the adhesive layer, parallel to the bonding line. The shear stress distribution curves for the three cross-sections are nearly symmetrical, with relatively uniform shear stress in the central region of the adhesive layer, distant from the interface end. Similar to the peel stress distribution, there is a pronounced concentration of shear stress at the left and right endpoints of the upper and lower interfaces of the adhesive layer. Additionally, it is observed that at the interior endpoints near the joint, the shear stress values approach zero (indicated by the red dotted line in Figure 15b. This indicates that the shear load on the joint is primarily supported by the central region of the adhesive layer, away from the endpoints. However, the shear stress in the adhesive layer reaches its peak near the interface ends, where significant stress concentrations in both shear and peel stress suggest that these areas are likely initial failure points in the joint structure. For the SikaPower^®^-1511 adhesive joint subjected to critical loading, the maximum principal stress distribution is illustrated in Figure 16.

In theory, for a joint with a center-symmetric structure, where the left end is fixed and the right end is subjected to a horizontal tensile load, the stress values of the upper and lower interface layers of the adhesive should be numerically equal at the center-symmetric position.

However, in the finite element simulation, minor asymmetries in boundary conditions and load application affect the complete symmetry of the stress distribution, resulting in numerical differences in peel and shear stresses at the two interfaces especially the ends of the joint. For instance, at the upper interface, the maximum peeling and shear stresses on the left side are $\sigma_{y}\;$= 52.635 MPa and $\tau_{xy}\;$= −13.381 MPa. Meanwhile, at the lower interface on the right side, the peel stress increases to a maximum of $\sigma_{y}\;$= 55.052 MPa, accompanied by a higher shear stress of $\tau_{xy}\;$= −15.043 MPa. This discrepancy in stress singularity is likely due to different force transmission paths in the numerical simulation but does not significantly impact the overall failure mode analysis of the adhesive joint.

Therefore, in the subsequent sections of this study, the lower interface—where the adhesive layer bonds with the adherend—and the midline within the adhesive layer are analyzed to elucidate the stress distribution and strength characteristics near the interface and within the adhesive layer for various resin epoxy adhesive combinations.

The stress distributions in epoxy adhesives are influenced by various factors, including the adhesive’s material properties and the applied external load, which directly shape the stress distribution within the adhesive layer. As the horizontal load increases, the interface’s singularity region exhibits complex stress evolution. To fully understand this behavior, comparing the adhesive layer’s stress response under moderate loading with its stress distribution near the critical load is crucial.

**Figure 17:**
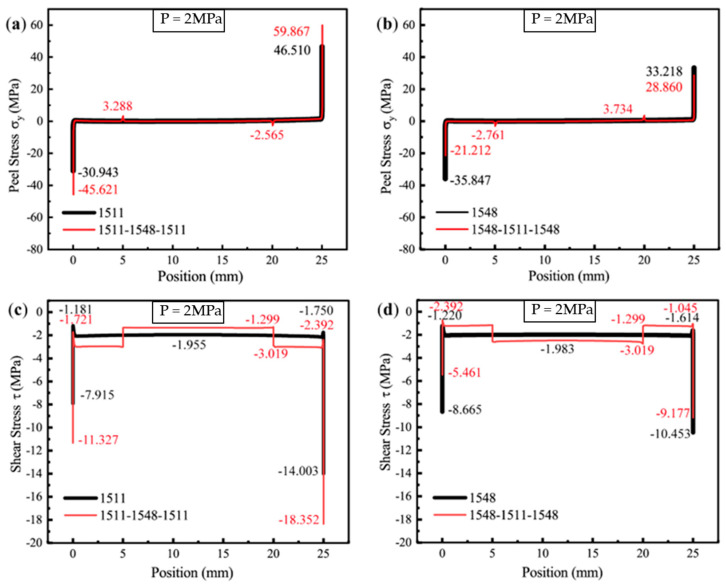
Stress distribution at the adhesive interface of different resin epoxy adhesive combinations under Pressure = 2 MPa. Peel stress ($\sigma_{y}$) for (**a**) 1511 and 1511-1548-1511 and (**b**) 1548 and 1548-1511-1548; Shear stress (τ) for (**c**) 1511 and 1511-1548-1511 and (**d**) 1548 and 1548-1511-1548.

Under a load of 2 MPa, the stress distribution primarily reflects the elastic response of the epoxy adhesive at the initial loading stage. Figure 17a,b illustrate the peel stress distributions at the adhesive interface for four adhesive configurations: SikaPower^®^-1511, SikaPower^®^-1548, 1511-1548-1511, and 1548-1511-1548. Similarly, the shear stress distributions at the interfaces of these adhesive configurations are shown in Figure 17c,d.

Analyzing the peel and shear stress distribution patterns across the four adhesive material combinations reveals a pronounced edge stress concentration at the ends of the bond. In the adhesive layer with a single adhesive, the stress values in the middle section are lower and exhibit greater stability. For the combined adhesive systems, a discontinuity in stress is observed at the transition regions between the two epoxy resins (5 mm and 20 mm), attributable to the differences in their mechanical properties. This is reflected in the stress distribution curves as a localized peak in peel stress at the transitions and a step-like change in shear stress. However, the shear stress in these regions remains relatively low, significantly below the stress peaks at the interface edges.

Since the absolute value of the stress curve is directly influenced by material properties and the applied load, when the load is constant and the pressure is maintained at 2 MPa (ensuring the material remains undamaged and the interface is in its initial elastic response stage), the extreme stress values at the interface edge stress concentration zones can be directly compared when the material combination at the bonding ends is fixed, utilized to compare the singular strength of the stress field for different joint configuration [68]. Specific theories and methods are as follows:

The generalized stress intensity factor (SIF), analogous to the classical SIF in linear elastic fracture mechanics, has been increasingly validated as a reliable fracture initiation parameter for dissimilar material joints. Recent studies have demonstrated that it effectively predicts the bond strength, which is governed by the SIF of the singular field at the interface end [69].

The singular stress field near the bonded joint interface ends is characterized by the singularity index λ, which can be calculated using Equation (1), which has two real roots for most material combinations.

(1) $$\begin{array}{c} 4\sin^{2}\left(\pi \lambda \right)\left\{\sin^{2}\left(\frac{\pi \lambda }{2}\right)-\lambda^{2}\right\}\beta^{2}+4\lambda^{2}\sin^{2}\left(\pi \lambda \right)\alpha \beta +\left\{\sin^{2}\left(\frac{\pi \lambda }{2}\right)-\lambda^{2}\right\}\alpha^{2}\\ -4\lambda^{2}\sin^{2}\left(\pi \lambda \right)\beta -2\left\{\lambda^{2}\cos\left(2\pi \lambda \right)+\sin^{2}\left(\frac{\pi \lambda }{2}\right)\cos\left(\pi \lambda \right)+\frac{1}{2}\sin^{2}\left(\pi \lambda \right)\right\}\alpha \\ +\sin^{2}\left(\frac{3\pi }{2}\lambda \right)-\lambda^{2}=0 \end{array}$$

α and β are Dundurs parameters [70], defined in terms of the Poisson’s ratio and shear modulus of the materials, where m = 1 represents the adhesive and m = 2 represents the adherend, shown in Equation (2).

(2) $$\alpha =\frac{G_{1}\left(\kappa_{2}+1\right)-G_{2}\left(\kappa_{1}+1\right)}{G_{1}\left(\kappa_{2}+1\right)+G_{2}\left(\kappa_{1}+1\right)},\;\beta =\frac{G_{1}\left(\kappa_{2}-1\right)-G_{2}\left(\kappa_{1}-1\right)}{G_{1}\left(\kappa_{2}+1\right)+G_{2}\left(\kappa_{1}+1\right)},\;\kappa_{m}=\{\begin{array}{c} \frac{3-v_{m}}{1+v_{m}}\;\;\left(\mathrm{plane}\;\mathrm{stress}\right)\\ 3-4v_{m}\;\;\left(\mathrm{plane}\;\mathrm{strain}\right) \end{array}\;\;\left(m=1,2\right)$$

The peel and shear stresses near the endpoint of the interface, as illustrated in Figure 14, are expressed as functions of the radial distance r from the singularity point O, as Equation (3):

(3) $$\sigma_{y}=\frac{K_{\sigma ,\lambda_{1}}}{r^{1-\lambda_{1}}}+\frac{K_{\sigma ,\lambda_{2}}}{r^{1-\lambda_{2}}}{,\;\tau }_{xy}=\frac{K_{\tau ,\lambda_{1}}}{r^{1-\lambda_{1}}}+\frac{K_{\tau ,\lambda_{2}}}{r^{1-\lambda_{2}}}$$

$K_{\sigma ,\lambda_{1}}$, $K_{\sigma ,\lambda_{2}}$ and $K_{\tau ,\lambda_{1}}$, $K_{\tau ,\lambda_{2}}$ are the stress intensity factors for the peel and shear stress fields, respectively, and $\lambda_{1}$, $\lambda_{2}$ denotes the singular index. The values of λ can be determined from Equation (1).

Equation (3) can be reorganized and further simplified to yield Equation (4), as shown below:

(4) $$\sigma_{y}=\frac{K_{\sigma ,\lambda_{1}}}{r^{1-\lambda_{1}}}+\frac{K_{\sigma ,\lambda_{2}}}{r^{1-\lambda_{2}}}≅\frac{K_{\sigma ,\lambda_{1}}}{r^{1-\lambda_{1}}}\left(1+C_{\sigma }r^{\lambda_{2}-\lambda_{1}}\right)\;\tau_{xy}=\frac{K_{\tau ,\lambda_{1}}}{r^{1-\lambda_{1}}}+\frac{K_{\tau ,\lambda_{2}}}{r^{1-\lambda_{2}}}≅\frac{K_{\tau ,\lambda_{1}}}{r^{1-\lambda_{1}}}\left(1+C_{\tau }r^{\lambda_{2}-\lambda_{1}}\right)\;C_{\sigma }=\frac{K_{\sigma ,\lambda_{2}}}{K_{\sigma ,\lambda_{1}}},C_{\tau }=\frac{K_{\tau ,\lambda_{2}}}{K_{\tau ,\lambda_{1}}}$$

Previous researchers’ studies have shown that the ratios $C_{\sigma }=\frac{K_{\sigma ,\lambda_{2}}}{K_{\sigma ,\lambda_{1}}}$ and $C_{\tau }=\frac{K_{\tau ,\lambda_{2}}}{K_{\tau ,\lambda_{1}}}$ remain nearly constant, except in cases involving extreme adhesive geometries [71].

Moreover, since $\lambda_{2}$ ≈ 1 [72], for models 1 and 2, which have different bond strength models but share the same joining angle (geometry) and fixed properties of the bonding material, and where the interface endpoints have the same singularity indices $\lambda_{1}$ and $\lambda_{2}$, a method is employed utilizing finite element analysis. This method approximates the ratio of stresses near the singularity to directly represent the ratio of the stress intensity factors in the vicinity of the singularity, as Equation (5) shows.

(5) $$\frac{\sigma_{\left(y,FEM\right)}^{1}}{\sigma_{\left(y,FEM\right)}^{2}}=\frac{K_{\sigma ,\lambda_{1}}^{1}}{K_{\sigma ,\lambda_{1}}^{2}},\frac{\tau_{\left(xy,FEM\right)}^{1}}{\tau_{\left(xy,FEM\right)}^{2}}=\frac{K_{\tau ,\lambda_{1}}^{1}}{K_{\tau ,\lambda_{1}}^{2}}$$

This method enables the direct assessment of the effect of graded adhesives in reducing the stress field intensity at the adhesive interface endpoint, without the need for explicitly calculating the strength factor at the site.

It is important to note that the application of this ratio method relies on the premise that Models 1 and 2 share the same stress field singularity index, $\lambda_{1}$, $\lambda_{2}$, near the endpoints of the bonding interface. For bonding combination models with identical material combinations at the endpoints—such as the 1511-1548-1511 model discussed in this paper and the model using only the Sikapower-1511 adhesive—the ratio of stress values at the interface endpoint can be directly interpreted as the ratio of the stress intensity factors at the same location.

For Figure 17a, the ratio $\sigma_{\left(y,1511\text{-}1548\text{-}1511\right)}^{1}/\sigma_{\left(y,1511\right)}^{2}$ = 59.867 MPa/46.510 MPa = 1.2872, indicates that transitioning from the 1511 configuration to the 1511-1548-1511 configuration results in a 28.72% increase in the peel singular stress field intensity.

For Figure 17c, at the left endpoint, the ratio $\tau_{\left(xy,1511\text{-}1548\text{-}1511\right)}^{1}/\tau_{\left(xy,1511\right)}^{2}$ = 18.352 MPa/14.003 MPa = 1.3106 corresponds to a 31.06% increase in the shear singular stress field intensity. At the right endpoint, the ratio $\tau_{\left(xy,1511\text{-}1548\text{-}1511\right)}^{1}/\tau_{\left(xy,1511\right)}^{2}$ = 11.327 MPa/7.915 MPa = 1.4311 demonstrates a 43.11% increase.

Figure 17a,c revealed that the edge peel and shear stress singularities in the joint structure of the 1511-1548-1511 adhesive combination were significantly higher than those observed in joints utilizing a single 1511 epoxy resin, indicating that the 1511-1548-1511 combination is more susceptible to localized damage under stress concentration, leading to earlier structural failure. This observation is consistent with finite element simulations, which predicted the lowest ultimate joint strength of 4.115 MPa among the four evaluated configurations.

Similarly, the model utilizing the 1548-1511-1548 adhesive has the same singularity index as the model using the 1548 adhesive alone, making the stress ratio an effective measure for representing the singular stress field intensity ratio.

For Figure 17b, the ratio $\sigma_{\left(y,1548\text{-}1511\text{-}1548\right)}^{1}/\sigma_{\left(y,1548\right)}^{2}$ = 28.860 MPa/33.218 MPa = 0.8688 indicates that transitioning from the 1548 configuration to the 1548-1511-1548 configuration results in a 13.12% decrease in the peel singular stress field intensity.

For Figure 17d, at the left endpoint, the ratio $\tau_{\left(xy,1548\text{-}1511\text{-}1548\right)}^{1}/\tau_{\left(xy,1548\right)}^{1}$ = 5.461 MPa/8.665 MPa = 0.6302 reflects a 36.98% decrease in the shear singular stress field intensity. Conversely, at the right endpoint, the ratio $\tau_{\left(xy,1548\text{-}1511\text{-}1548\right)}^{1}/\tau_{\left(xy,1548\right)}^{1}$ = 9.177 MPa/10.453 MPa = 0.8779 shows a 12.21% indicates a 12.21% decrease in the shear singular stress field intensity.

The 1548-1511-1548 adhesive material combination demonstrated an effective reduction in the peel and shear stress singularities at the bonded ends of the joint interface. This configuration significantly improved the joint’s load-bearing capacity, with simulations predicting an ultimate strength of 10.941 MPa.

For models with fixed adhesive materials, the edge stress singularities provide a direct basis for evaluating the performance of the adhesive material of the joint. The differing effects of stress singularity magnitudes for joint strength between the two combined adhesive configurations underscore the critical importance of selecting appropriate adhesive material combinations in the design of gradient adhesives.

Under critical conditions, although the differences in the horizontal loads applied prevent a direct assessment of the joint strength by simply comparing the numerical values of the stress curves, it remains essential to extract the stress distribution characteristics of the adhesive layer in the critical failure state. By analyzing the complex stress response in the adhesive layer of the epoxy adhesive at the dangerous regions of the joint, valuable insights can be obtained to predict the initiation location of the adhesive failure and to identify the potential failure modes.

**Figure 18:**
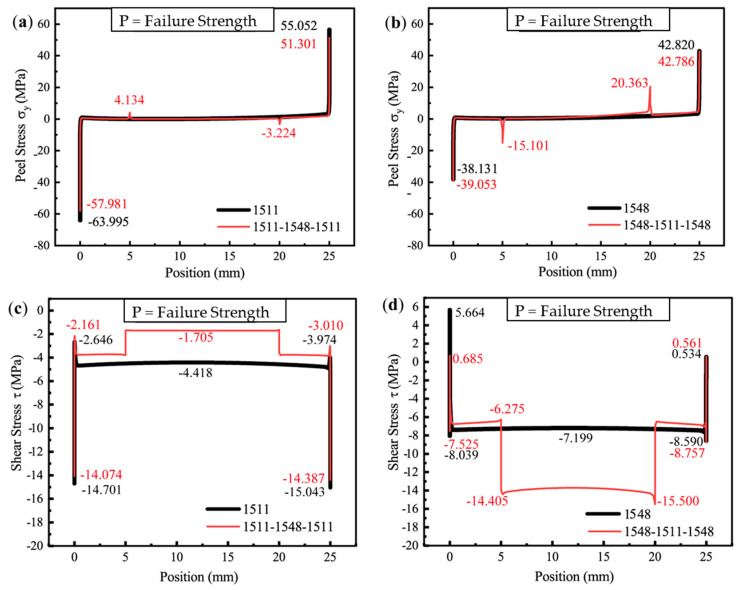
Stress distribution at the adhesive interface of different resin epoxy adhesive combinations under Pressure = Failure Strength. Peel stress ($\sigma_{y}$) for (**a**) 1511 and 1511-1548-1511 and (**b**) 1548 and 1548-1511-1548; Shear stress (τ) for (**c**) 1511 and 1511-1548-1511 and (**d**) 1548 and 1548-1511-1548.

When the applied load reaches the critical strength of each model, the peel stress distribution patterns on the interfaces of the four different adhesive combinations are presented in Figure 18a,b, while the shear stress distribution patterns are shown in Figure 18c,d.

**Figure 19:**
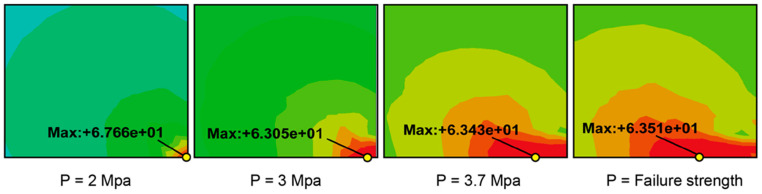
The stress redistribution under an increasing load of SikaPower^®^-1511 adhesive.

Under the critical ultimate load, the stress distribution of adhesive peeling at the interface ends exhibits a similar pattern to that observed under a pressure of 2 MPa (Figure 17): The stress in the middle region, away from the adhesive edges, remains relatively low, while significant stress concentration occurs at the interface ends. As shown in Figure 17a and Figure 18a, the adhesive 1511 reaches a peak singular stress value of 55.052 MPa near the right endpoint of the interface, which exceeds the corresponding value of 46.510 MPa at 2 MPa. In contrast, for the 1511-1548-1511 adhesive combination, the extreme peel stress at the interface end node decreases from 59.867 MPa to 51.301 MPa. This reduction can be attributed to the stronger singular stress field present at the interface corner in this combination, which induces premature localized failure due to stress concentration. This failure results in more complex micro-scale damage in the region, prompting stress redistribution illustrated in Figure 19.

The phenomenon of stress redistribution in Figure 19. demonstrates that under critical load conditions, the material near the singularity exhibits a more intricate stress response, while the location of the maximum principal stress shifts from the corner to adjacent units, partially alleviating the stress concentration at the boundary corner.

For the two-component epoxy adhesive, a secondary peak in the singular peel stress is consistently observed in the transition region between the two materials. However, the order of application leads to distinct differences in the magnitude of this secondary peak. For instance, in the adhesive configuration 1548-1511-1548, the peel stress at the material property discontinuity (20 mm) increases significantly from 3.734 MPa (Figure 17b) to 20.363 MPa (Figure 18b) under the critical load. In comparison, for the adhesive combination 1511-1548-1511, the extreme peel stress at 5 mm exhibits a much smaller increase, rising only from 3.288 MPa to 4.134 MPa as the load increases from 2 MPa to the critical value.

The shear stress distribution under the critical load visually illustrates the load-bearing behavior of the adhesive at the ultimate shear load. Compared to the elastic response stage at the initial loading condition (Pressure = 2 MPa), the stress distribution curve exhibits a marked difference.

As shown in Figure 18c, the shear stress-bearing capacity of the adhesive combination 1511-1548-1511 (red line) is significantly lower than that of the single 1511 adhesive (black line). Under the critical load, the shear stress in the middle section of the 1511-1548-1511 combination (corresponding to the region of the 1548 epoxy resin, i.e., 5–20 mm) is only −1.705 MPa. This indicates that the tough 1548 adhesive in the middle section fails to effectively bear the main load. Consequently, a substantial portion of the load is redistributed to the stiffer 1511 adhesive at both ends (0–5 mm and 20–25 mm). The high stress concentration at the interface corners of this combined configuration further reduces the shear load capacity of the 1511 adhesive sections compared to a joint using a single 1511 adhesive. As a result, the inefficient load distribution ultimately diminishes the overall load-bearing capacity.

Figure 18d presents a shear stress distribution pattern distinctly different from that in Figure 17d, with the shear stress borne by the middle section of the adhesive layer significantly exceeding that observed under a loading pressure of 2 MPa, as the shear stress borne by the middle of the 1548 adhesive increases from −1.983 MPa to −7.199 MPa. For adhesives with a 1548-1511-1548 configuration, the shear load is primarily carried by the middle section of the adhesive layer (5 mm–20 mm), where the 1511 epoxy resin is located. The step effect, resulting from the abrupt change in shear stress due to the discontinuous material properties, becomes more pronounced. At the transition points between the two materials, the shear stress can reach values of −14.405 MPa and −15.500 MPa.

This configuration effectively utilizes the load-bearing capacity of the rigid 1511 epoxy resin located in the middle, while the more ductile 1548 material at the ends of the gradient adhesive helps to reduce stress concentration at the interfaces. Such a reasonable adhesive combination achieves high utilization of the rigid epoxy resin’s load-bearing capacity, highlighting the potential of functionally graded adhesives in enhancing the shear resistance of joints.

**Figure 20:**
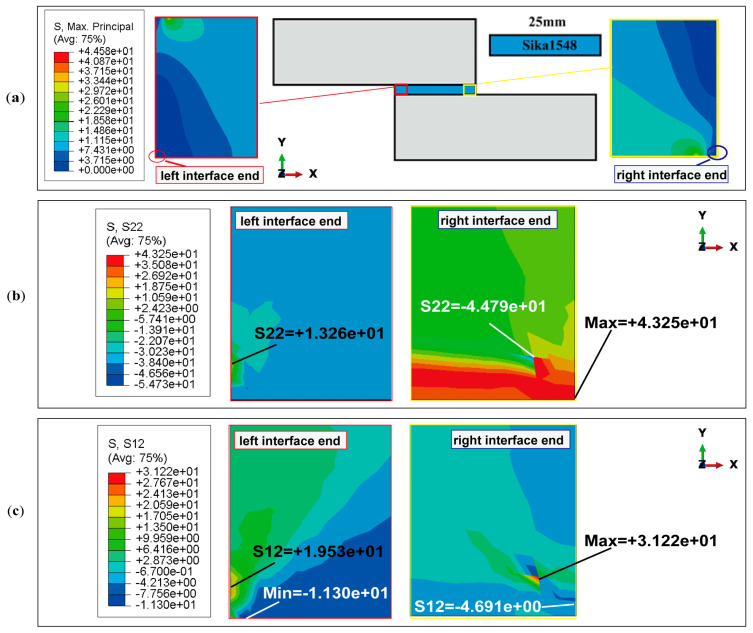
The complex stress states near the two interface ends under critical damage conditions of SikaPower^®^-1548 adhesive. (**a**) Max Principal Stress. (**b**) Peel Stress $\sigma_{y}$($S_{22}$). (**c**) Shear Stress $\tau_{\mathrm{xy}}$($S_{12}$).

It is worth noting that the shear stress of the 1548 adhesive exhibits a singularity jump at the left end of the interface, rising from −8.039 MPa to 5.664 MPa, as shown in Figure 18d. For tough adhesives with strong plastic deformation capacity, the stress response of elements near the interface under critical load becomes more complex due to plastic deformation, compared to the yielding behavior of the brittle 1511 epoxy resin with limited ductility. The peel stress and shear stress distributions near the interface end of the epoxy resin SikaPower^®^-1548 adhesive under initial loading and at the critical ultimate load are illustrated in Figure 20.

**Figure 21:**
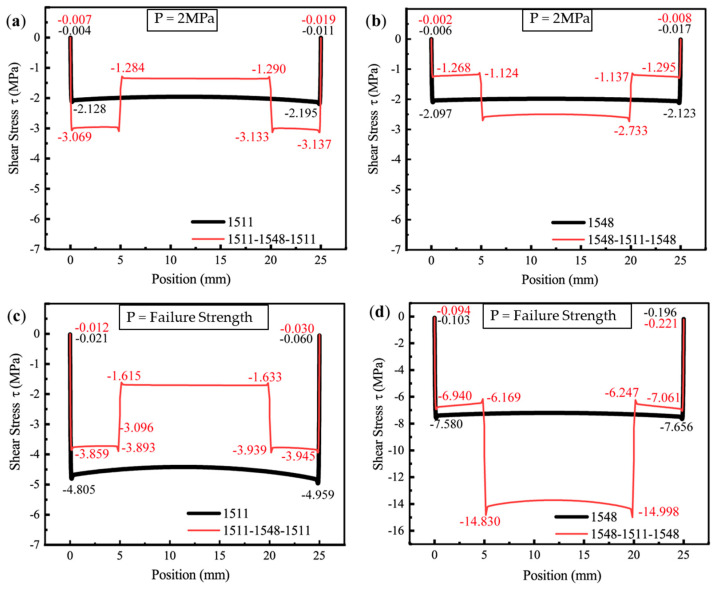
Shear stress distribution at the adhesive middle line of different resin epoxy adhesive combinations under Pressure = 2 MPa for (**a**) 1511 and 1511-1548-1511 and (**b**) 1548 and 1548-1511-1548;.and under Pressure = Failure Strength for (**c**) 1511 and 1511-1548-1511 and (**d**) 1548 and 1548-1511-1548.

Since the peel stress along the middle line in the thickness direction of the adhesive layer remains close to zero, as Figure 15a shows, that only the shear stress distribution within the adhesive layer is investigated, as shown in Figure 21.

Under an applied load of 2 MPa, in Figure 21a,b, the shear stress in the four models was found to remain at a low level, with a relatively flat and uniform distribution. Moreover, the stress step effect in the discontinuous region between the two materials of the gradient adhesive is not significant. By contrast, under a critical load, the central layer shear stress of the four adhesives increases, showing an overall rising trend.

As shown in Figure 21c, the 1511-1548-1511 adhesive combination, marked by the red line, fails to fully utilize the material’s shear-bearing capacity. Notably, for the joint employing only the 1511 adhesive, the shear stress distribution curve is less uniform compared to the model using solely the 1548 adhesive in Figure 21d. The shear stress in the central region of the 1511 adhesive is slightly lower than the extreme values of −4.805 MPa and −4.959 MPa observed near the left and right ends, indicating that the load-bearing capacity of the rigid epoxy resin 1511 in the middle segment has not been fully realized at this stage. At the same time, it was found that for the shear stress curve of the 1548-1511-1548 adhesive combination, the load-bearing capacity of the 1511 adhesive in the middle section is significantly enhanced. However, a pronounced tendency for stress concentration arises at the material discontinuities of the combined adhesive. Additionally, the shear stress in the middle section (around 12.5 mm) remains lower than the values of −14.830 MPa near 5 mm and −14.998 MPa near 20 mm. This indicates that further improvement and optimization are required to enhance load transfer capability, mitigate stress singularities, and improve the overall performance of gradient joint designs using adhesive materials with differing mechanical properties.

**Figure 22:**
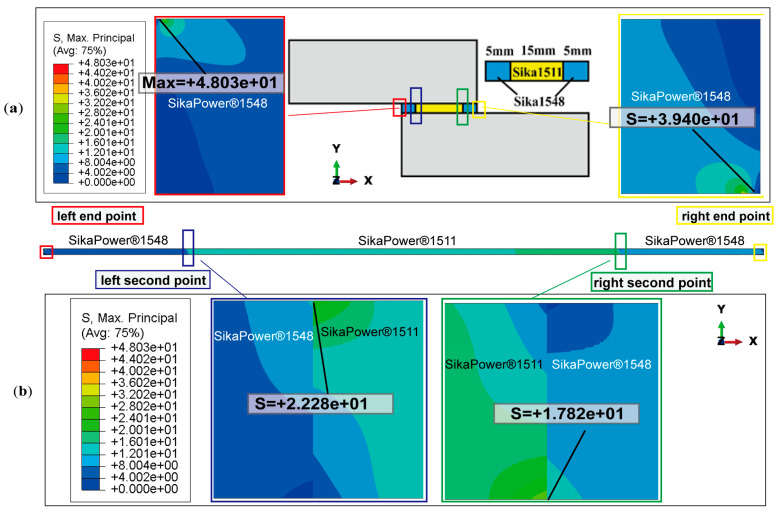
Max Principal Stress distribution of 1548-1511-1548 adhesive combinations under Pressure = Failure Strength. (**a**) at the adhesive ends. (**b**) at the second dangerous positions.

Furthermore, for a gradient adhesive composed of two epoxy resin formulations, the discontinuous stress at the interface, arising from the differing mechanical properties of the materials, represents the second most critical failure risk after the interface end. Stress singularities in this region are significant considerations in the design of functionally graded joints. However, in this study, under the critical load, the elements near this second critical location have not yet reached the material’s damage threshold (Figure 22). Instead, cohesive failure initiates near the endpoint of the interface, where the adhesive is bonded to the substrate.

## 5. Discussion

In the curing of epoxy resins, empirical models have always been used to study curing kinetics. One such model is the Kamal model [73], which assumes two distinct curing mechanisms. The first mechanism, known as nth order, occurs from the start of the process up to approximately 20% conversion. During this interval, the oxirane ring opens, producing OH groups that autocatalyze the reaction, driving it forward. The second mechanism is autocatalytic, occurring from 20% to about 80–85% curing. Because it is autocatalytic, this stage requires less activation energy than the initial phase. At the end of the reaction, when few OH groups and amines remain, along with the steric effects of cross-linking, an additional supply of energy is needed to complete the curing, and the reaction returns to order n. This model yields two reaction constants, with the nth order constant (k_1_) typically being lower than the autocatalytic constant (k_2_).

Another model commonly used is the Kissenger model [74], which assumes the entire curing process follows nth order, with n = 1. This model is faster but less precise. Finally, there is the Free Kinetic Model (MFK) developed by Vyazovkin [75], which assumes that the activation energy of the process changes with the degree of conversion. This model is empirical and more similar to the Kamal model, and it has been incorporated into some software tools. These mechanisms have already been used by the research group at Carlos III University of Madrid to study the curing kinetics of epoxy resins, with outstanding results in this field [76,77,78].

In this study, the MFK model was used to calculate the activation energy (E_a_, Figure 6) of the curing process. This E_a_ varies from one adhesive to another and depends fundamentally on the epoxy composition. Figure 5 shows that SikaPower^®^-1548, due to its composition (component A: bisphenol-A-epichlorohydrin and epoxy resins; component B: N′-(3-aminopropyl)-N,N-dimethylpropane-1,3-diamine and 1,8-diamino-3,6-diazaoctane), aligns well with the Kamal model. However, at the end of the reaction, the E_a_ does not increase, which is consistent with less cross-linking and, consequently, a more ductile adhesive.

In contrast, for SikaPower^®^-1511, the initial reaction requires a relatively low E_a_ to start. This effect is likely due to the presence of mercaptan in component B, which accelerates the curing reaction and facilitates high cross-linking in a short time (Figure 5a). Mercaptans contain an active hydrogen in an S-H group, meaning polymercaptans have multiple active hydrogen atoms. This curing process requires a tertiary amine accelerator, such as 2,4,6-tris (dimethylaminomethyl) phenol. By adjusting the amount of accelerator, the gelation time can be controlled, enabling faster cures, which aligns with this adhesive’s formulation [79].

The two curing peaks observed in SikaPower^®^-1511 (Figure 4) and the broader curing peak in SikaPower^®^-1548 are associated with component A, where multiple epoxy resins are combined in addition to bisphenol A and epichlorohydrin.

The infrared study confirmed the presence of C-SH groups and phenols in the SikaPower^®^-1511 adhesive (Figure 7a and Table 4), while amines are more predominant in SikaPower^®^-1548 (Figure 7b and Table 4).

The relatively low initial T_g_ suggests limited cross-linking of the resin chains at this early stage. It appears that SikaPower^®^-1511 may require additional energy in its final stage to achieve full curing. Thus, although it cures quickly, it reaches its maximum strength over a longer period, aligning with the increased cross-linking necessary for full performance. Therefore, for SikaPower^®^-1548 although its curing process takes longer, it is likely that the reaction will proceed more thoroughly, leading to a more complete cure in this adhesive.

The two glass transition temperatures (T_g_) found in the DSC scans of the cured epoxies confirm the presence of two types of epoxy resins in each adhesive (Table 5). Additionally, infrared analysis (Figure 8) indicates that approximately 5% of the resin in SikaPower^®^-1511 remains uncured, which shifts the second T_g_ from 94 °C to 140 °C (Table 5). This partial curing increases the resin’s toughness by reducing cross-linking density. High-temperature post-curing processes could enhance the adhesive’s rigidity.

The values obtained in the shear tests are relatively low compared to those in the tensile tests, and there is little correlation between them, particularly in terms of strain percentage, as SikaPower-1548 remains the most ductile. Specifically, the adhesive with the highest tensile strength (SikaPower^®^-1511, Figure 9 and Table 6) exhibits the lowest shear strength (Figure 10 and Table 7). This discrepancy may be related to the adhesive’s bonding with the steel substrate. It’s possible that the more ductile adhesive, which takes longer to begin curing (SikaPower^®^-1548, Figure 5b and Figure 9, and Table 6), wets the steel surface more effectively. In contrast, the stiffer, higher-strength adhesive (SikaPower^®^-1511), which cures very quickly (Figure 5a), may wet the steel surface less effectively, as it has minimal time to establish good contact before cross-linking begins. Although both adhesives exhibit adhesive failure upon breaking, SikaPower^®^-1548 demonstrates better adhesion performance.

The macroscopic understanding of adhesive bond strength is typically measured through mechanical testing, with the adhesive shear test being the most common method. Additionally, the failure mode of the adhesive can be analyzed by examining the microscopic structure of the adhesive fracture surface post-experiment, using techniques such as scanning electron microscopy (SEM). Beyond these traditional macroscopic methods, some researchers have delved into the molecular-level failure modes of adhesives. For instance, Tsujiet al. [80] developed a molecular understanding of cohesive failure by studying the system of epoxy resin bonded to a hydroxyl-terminated self-assembled monolayer (SAM) surface, offering a new perspective on adhesive failure modes. Miyata et al. [81] investigated the adhesive interface between the epoxy resin and a silicon substrate, studying the impact of the surface chemistry of the inorganic material on the structure of the epoxy resin and the fracture behavior near the adhesive interface. It was proposed that within a few nanometers of the adhesive interface, the cross-linking structure of the epoxy resin and the molecular adsorption structure at the interface are influenced by the intermolecular dynamics of the inorganic material, which in turn affects the fracture mode and adhesion strength of the adhesive. This approach offers a novel perspective on adhesive strength analysis.

In finite element simulations of adhesive joints, various failure criteria are commonly employed to predict the ultimate load of the bond. This study analyzed four different lap-joint models based on the mechanical properties of two epoxy resins measured in previous experiments. The ductility of the adhesive material near the end of the adhesive layer (i.e., the potential first failure point) was considered to distinguish the failure behavior. The Critical Distance Method (CDM) was applied to predict local failure at the interface corner near the epoxy resin. For the rigid adhesive SikaPower^®^-1511, the maximum principal stress criterion was used. In contrast, for the more ductile adhesive SikaPower^®^-1548, a ductile fracture criterion accounting for plastic deformation and local strain accumulation was adopted. Finite element simulations provided reasonable predictions of the strength of single adhesive joints, as shown in Table 10. However, discrepancies between simulation and experimental results were observed in the strength predictions of graded adhesives. These differences were analyzed regarding the idealized assumptions of finite element modeling, the complex thermal curing behavior of graded adhesives, and potential interactions between the two epoxy resins. Despite these discrepancies, the overall good agreement between simulation and experimental results validates the use of distinct damage fracture criteria to predict the bond strength of adhesives with differing mechanical properties.

Due to the complex and different failure modes of adhesive joints, damage patterns can differ significantly under varying load conditions, adhesive properties, and joint geometries. Mixed failure modes involving multiple damage mechanisms are also possible. Consequently, finite element simulation is crucial for modeling and analyzing stress singularities and damage-prone regions, particularly in studies of graded adhesive structural failure. Most existing research on stress distribution within adhesive focuses on the middle layer [55,82], with relatively little attention paid to stress distribution at adhesive interfaces. In this study, using SikaPower^®^-1511 as an example, Figure 15 illustrates the peel and shear stress distributions in the adhesive interface layer and the middle adhesive layer, highlighting their distinct stress distribution. Subsequent analyses compare the stress responses of the interface layer and the middle layer under various loading conditions, investigating how gradient adhesives mitigate stress singularities at adhesive interface ends (Figure 17 and Figure 18). Additionally, the stress distribution of the middle adhesive layer under critical load conditions (Figure 21) demonstrates the potential of gradient adhesives to enhance shear load-bearing efficiency.

For epoxy adhesives, detailed stress analysis near the primary failure point (Figure 16 and Figure 20) is essential for verifying failure predictions across different adhesive configurations. When the adhesive layer interface end is bonded with rigid epoxy resin, stress singularities at the corner often lead to premature failure, resulting in similar and relatively lower shear strengths for the SikaPower^®^-1511 and 1511-1548-1511 models. In contrast, more ductile adhesives exhibit greater plastic deformation at the adhesive interface ends, leading to higher and comparable shear strengths in the SikaPower^®^-1548 and 1548-1511-1548 models.

In addition, the design of gradient adhesives introduces stress discontinuities due to the discontinuity of mechanical properties between the two epoxy resins (Figure 22), often resulting in the formation of a potential secondary failure point within the adhesive layer. While the adhesive failure in this study still initiates at the interface end, the evaluation of this secondary failure location is critical and should not be overlooked in graded adhesive design.

For gradient adhesives utilizing epoxy resin materials, a promising research direction lies in improving the uniformity of stress distribution along the adhesive bond length. Future efforts should focus on selecting two or more epoxy resin adhesives with compatible curing characteristics, chemical properties, and mechanical properties, and strategically integrating them within functional adhesive layers. Such advancements aim to achieve uniform stress distribution, optimize load-bearing efficiency, and unlock greater potential in the design of gradient adhesives for advanced engineering applications.

In addition, future lines of research will prioritize the implementation of an automatic adhesive application system to achieve more precise and reproducible joints. Furthermore, alternative surface preparation methods for steel are being tested to enhance the wettability of this type of adhesive, which could further improve joint performance and reliability.

## 6. Conclusions

Two epoxy resins, SikaPower^®^-1511 and SikaPower^®^-1548, were analyzed for their curing kinetics and tensile strengths. SikaPower^®^-1511 cures quickly at room temperature (90–95%) due to the use of mercaptan as a hardener, but this rapid process results in incomplete curing, limiting its toughness. In contrast, SikaPower^®^-1548 cures more slowly but achieves a more complete reaction, making it approximately 31% tougher.

In terms of tensile properties, SikaPower^®^-1511 shows higher tensile strength and stiffness, classifying it as a rigid adhesive. However, SikaPower^®^-1548 outperforms in shear strength, with 9 MPa compared to 4 MPa for SikaPower^®^-1511. This difference is attributed to the slower curing of SikaPower^®^-1548, which allows better wetting of the steel substrate, while the rapid curing of SikaPower^®^-1511 reduces its ability to establish good surface contact.

Although both adhesives exhibit adhesive failure, SikaPower^®^-1548 demonstrates superior adhesion performance. This is linked to its cross-linking structure and the intermolecular dynamics at the interface, which enhance its fracture behavior and adhesion strength.

Simultaneously with the experiments, a simulation of the adhesive joints was conducted. The simulation focused on analyzing the stress distribution and failure behavior of adhesive joints under various loading conditions. Finite element modeling was employed to evaluate the performance of both rigid and ductile adhesives, as well as combined configurations, providing insights into their mechanical response and failure mechanisms.

SikaPower^®^-1511, a rigid adhesive, uses a maximum principal stress criterion, while the ductile SikaPower^®^-1548 follows a plastic deformation-based criterion. Simulations aligned well with experiments for single adhesives but showed discrepancies for graded models (1511-1548-1511 and 1548-1511-1548) due to idealized modeling and curing complexities. Combined models demonstrated that 1548-1511-1548 achieves higher shear strength due to greater ductility, while 1511-1548-1511 exhibits lower strength due to stress singularities at interface corners. Gradient adhesives enhance shear load efficiency and mitigate stress concentrations.

Overall, the combination of experimental and simulation approaches provides valuable insights into the distinct mechanical behaviors of SikaPower^®^-1511 and SikaPower^®^-1548. These findings underscore the potential of gradient adhesive systems to optimize joint performance under varying loading conditions.

## Figures and Tables

**Table 1:** Chemical composition and mechanical properties of steel AISI 4140.

**Composition**	**C**	**Si (≤)**	**Mn (≤)**	**P (≤)**	**S (≤)**	**Cr**	**Mo**
	0.38–0.43	0.15–0.35	0.75–1.00	0.035	0.04	0.80–1.10	0.15–0.25
**Mechanical Properties**	**Tensile strength (MPa) ≥**	**Yield strength (MPa) ≥**	**Elongation in 50 mm, %, ≥**	**Reduction in area, % ≥**		**Hardness (HB)**	
	1020	655	17.7	46.8		302	

**Table 2:** Peak temperature and Enthalpy of curing at different rates for SikaPower^®^-1511 and SikaPower^®^-1548 adhesives.

SikaPower^®^-1511				SikaPower^®^-1548	
Heating Rate (°/min)	Peak Temperature (°C)		DH_c_ (J/g)	Peak Temperature (°C)	DH_c_ (J/g)
	1st Peak	2nd Peak			
5	36 ± 1	102 ± 1	202 ± 1	101 ± 2	300 ± 2
10	51 ± 1	112 ± 2	203 ± 3	116 ± 1	295 ± 4
15	68 ± 1	117± 1	205 ± 2	123 ± 1	299 ± 2
20	86 ± 1	120 ± 1	203± 2	129 ± 3	297 ± 3

**Table 3:** Curing process simulation at different temperatures for SikaPower^®^-1511 and SikaPower^®^-1548.

Adhesive	Degree of Conversion (%)	Temperature (°C)				
		25	30	50	75	100
		Time (min)				
SikaPower^®^-1511	5	1.8	1.4	0.5	0.2	0.1
	20	4.0	3.2	1.5	0.6	0.3
	40	6.1	5.2	2.8	1.5	0.8
	70	8.5	7.4	4.3	2.4	1.5
	90	41.1	33.0	14.6	6.1	2.8
	99	750.3	523.0	123.4	25.4	6.4
SikaPower^®^-1548	5	67.0	39.1	5.4	0.6	0.3
	20	136.8	92.5	21.8	4.5	1.2
	40	229.3	159.3	41.6	9.7	2.7
	70	323.8	232.8	68.9	18.3	5.8
	90	423.2	311.9	101.1	29.7	10.3
	99	715.4	533.4	180.5	55.5	20.0

**Table 4:** Assignment of functional groups based on vibrations observed in the FTIR spectra for Components A and B of the adhesives SikaPower^®^-1511 and SikaPower^®^-1548 [66].

Wavenumber (cm^−1^)	Functional Groups/Vibration	Components	
		A	B
723	C-H out of plane deformation/bending		1548
765		1548/1511	
827		1548/1511	1511
914	C-O-C oxirane	1548/1511	
1014	Primary amine C-N		1548
1030	C-O-C stretching (aromatic)	1548/1511	
1080			1511
1100	C-H deformation—phenols		1511
1120	H-C-H Bending	1548	1548
1182		1548/1511	
1245	C-O-C stretching/ N-H deformation	1548/1511	1548
1290			1548/1511
1367	CH-S deformation		1511
1450	C-O stretching	1458/1511	
1456	Tertiary amines		1548/1511
1508	C-C aromatic ring	1548/1511	
1556	N-H secondary deformation		1548
1606	C=C stretching Phenols C=C	1548/1511	1548
1650			1548/1511
1730	C=O stretching	1548	
2750–3000	C-H stretching	1548/1511	1548/1511
3000–3400	NH stretching	1548/1511	1548/1511
3000–4000	C=N/C=N-OH		1511

**Table 5:** Glass transaction temperatures for SikaPower^®^-1511 and SikaPower^®^-1548.

Adhesive	1st Scan		2nd Scan	
	T_g1_ (°C)	T_g2_ (°C)	T_g1_ (°C)	T_g2_ (°C)
SikaPower^®^-1511	38 ± 2	94 ± 1	37 ± 1	140 ± 1
SikaPower^®^-1548	70 ± 1	127 ± 1	72 ± 1	129 ± 1

**Table 6:** Summary of mechanical properties for SikaPower^®^-1511 and SikaPower^®^-1548 adhesives, including maximum tensile stress, strain at maximum stress, Young’s Modulus, and Shore D hardness.

Adhesive	σ_max_ (MPa)	ε (%) in σ_max_	Young’s Modulus (MPa)	Hardness (SDH)
SikaPower^®^-1511	51 ± 10	11 ± 1	490 ± 60	80 ± 1
SikaPower^®^-1548	33 ± 3	16 ± 3	270 ± 50	72 ± 2

**Table 7:** Shear strength and Strain for SikaPower^®^-1548, SikaPower^®^-1511 and graduated joints, with SikaPower^®^-1548 on the edges (1548-1511-1548) and SikaPower^®^-1511 on the edges (1511-1548-1511).

Adhesives	Shear Strength (MPa)	Strain (%)
SikaPower^®^-1511	4 ± 2	6 ± 2
SikaPower^®^-1548	9 ± 3	11 ± 2
1548-1511-1548	8 ± 2	9 ± 1
1511-1548-1511	5 ± 1	6 ± 2

Note: Shear strength and strain values are averages from five specimens, with errors as standard deviations.

**Table 8:** Mechanical properties of SikaPower^®^-1511 and SikaPower^®^-1548.

Property	SikaPower^®^-1511	SikaPower^®^-1548
Young’s modulus, $E_{a}$ [MPa]	490	270
Poisson’s ratio, $\nu_{a}$	0.367	0.396
Tensile strength, $\sigma_{fa}$ [MPa]	51	33
Elongation at break, $\varepsilon_{fa}$ [%]	3	9
Plastic hardening modulus, $E_{plastic}$ [MPa]	165	50
Shear Modulus, G [MPa]	179.3	96.7

**Table 9:** Finite element mesh sensitivity analysis of peel stress (σ_y_) at various nodes near the adhesive interface endpoint.

Node	Position (mm)	Peel Stress σ_y_ (MPa)				Ratio = σ_0.2_/σ_0.1_
		E_min_ = 0.1 μm	E_min_ = 0.2 μm	E_min_ = 0.5 μm	E_min_ = 1 μm	
1	0	37.346	31.413	24.598	20.483	0.8411
2	0.0005	12.214	12.343	11.770	13.939	1.0106
3	0.001	10.087	10.081	10.234	9.649	0.9995
4	0.0015	8.989	8.990	8.961	8.721	1.0002
5	0.002	8.275	8.272	8.271	8.400	0.9996
6	0.0025	7.742	7.743	7.737	7.775	1.0002
7	0.003	7.325	7.328	7.320	7.295	1.0004
8	0.0035	6.979	6.982	6.976	6.956	1.0004
9	0.004	6.685	6.687	6.684	6.682	1.0002
10	0.0045	6.432	6.433	6.430	6.424	1.0002
11	0.005	6.205	6.205	6.206	6.200	0.9999

Note: This table presents the peel stress values (σ_y_) at nodes along the adhesive interface under a horizontal load of 2 MPa. The nodes are numbered sequentially starting from the singular stress point at the corner of the adhesive (SikaPower^®^-1511)-substrate (steel) interface. The corresponding positions represent their radial distance (Position) from this corner. The analysis evaluates results for minimum mesh sizes (E_min_) of 0.1 μm, 0.2 μm, 0.5 μm, and 1 μm. The ratio of peel stresses (σ_0.2_/σ_0.1_) quantifies the convergence accuracy and assesses the mesh independence of the finite element simulation.

**Table 10:** Simulation result and experiment result.

Adhesives	Simulation Failure load (KN)	Simulation Strength (MPa)	Experimental Strength (MPa)
SikaPower^®^-1511	2.82	4.515	4 ± 2
SikaPower^®^-1548	5.17	8.274	9 ± 3
1511-1548-1511	2.57	4.115	5 ± 1
1548-1511-1548	6.84	10.941	8 ± 2

## Data Availability

The raw data supporting the conclusions of this article will be made available by the authors on request.

## References

[1] Karthikeyan N., Naveen J. (2024). Progress in adhesive-bonded composite joints: A comprehensive review. J. Reinf. Plast. Compos..

[2] Wei Y., Jin X., Luo Q., Li Q., Sun G. (2024). Adhesively bonded joints–A review on design, manufacturing, experiments, modeling and challenges. Compos. Part B.

[3] Borges C.S.P., Akhavan-Safar A., Tsokanas P., Carbas R.J.C., Marques E.A.S., da Silva L.F.M. (2023). From fundamental concepts to recent developments in the adhesive bonding technology: A general view. Discov. Mech. Eng..

[4] Desai C.R., Patel D.C., Desai C.K. (2023). Investigations of joint strength & fracture parameter of adhesive joint: A review. Mater. Today Proc..

[5] Jeevi G., Nayak S.K., Abdul Kader M. (2019). Review on adhesive joints and their application in hybrid composite structures. J. Adhes. Sci. Technol..

[6] Schlechte J.S. (2023). Advances in Structural Adhesive Bonding.

[7] Zhou M.-H., Yin G.-Z., Prolongo S.G. (2024). Review of thermal conductivity in epoxy thermosets and composites: Mechanisms, parameters, and filler influences. Adv. Ind. Eng. Polym. Res..

[8] Sun S., Yu Q., Yu B., Zhou F. (2023). New Progress in the Application of Flame-Retardant Modified Epoxy Resins and Fire-Retardant Coatings. Coatings.

[9] Makhmetova A., Negim E.-S., Ainakulova D., Yeligbayeva G., Khatib J. (2024). An Overview of Epoxy Resins as coating to protect metals from corrosion. Complex Use Miner. Resour..

[10] Faggio N., Marotta A., Ambrogi V., Cerruti P., Gentile G. (2023). Fully bio-based furan/maleic anhydride epoxy resin with enhanced adhesive properties. J. Mater. Sci..

[11] Zhang K., Wang Z., Luo Y. (2024). One-component epoxy resin adhesive featured with high storage stability based on microencapsulation. Colloids Surf. Physicochem. Eng. Asp..

[12] La L.B.T., Nguyen H., Tran L.C., Su X., Meng Q., Kuan H.-C., Ma J. (2024). Exfoliation and dispersion of graphene nanoplatelets for epoxy nanocomposites. Adv. Nanocompos..

[13] Khalid M.Y., Kamal A., Otabil A., Mamoun O., Liao K. (2023). Graphene/epoxy nanocomposites for improved fracture toughness: A focused review on toughening mechanism. Chem. Eng. J. Adv..

[14] Białkowska A., Bakar M., Kucharczyk W., Zarzyka I. (2023). Hybrid epoxy nanocomposites: Improvement in mechanical properties and toughening mechanisms—A review. Polymers.

[15] Ba H., Guo L., Huan H., Zhang S., Lin Z. (2023). Multi-objective optimization of epoxy resin adhesive for pavement toughened by self-made toughening agent. Polymers.

[16] Huo M., Chen J., Jin C., Huo S., Liu G., Kong Z. (2024). Preparation, characterization, and application of waterborne lignin-based epoxy resin as eco-friendly wood adhesive. Int. J. Biol. Macromol..

[17] Li A., He H., Shen Y., Li Q. (2023). High peel strength and excellent solder heat-resistance epoxy adhesive for flexible copper clad laminate. J. Appl. Polym. Sci..

[18] Cherian R.M., Unnikrishnan T.G., Cherian M., Joy J., Chirayil C.J., Panneerselvam K., Thomas S. (2024). Chapter 11—Epoxy resins: Synthesis, structure, and properties. Handbook of Thermosetting Foams, Aerogels, and Hydrogels.

[19] Huang C., Sun X., Yuan H., Song C., Meng Y., Li X. (2020). Study on the reactivity and kinetics of primary and secondary amines during epoxy curing by NIR spectroscopy combined with multivariate analysis. Vib. Spectrosc..

[20] Puhurcuoğlu N., Arman Y. (2024). Parameter estimation of epoxy resin cure kinetics by dynamics DSC data. Polym. Adv. Technol..

[21] Chu C.-W., Cheng C.-H., Obayashi K., Bayomi R.A.H., Takahara A., Kojio K. (2024). Effects of curing conditions on adhesive and fatigue properties of hydrogenated epoxy resins in bulk state and single-lap-joint configuration. Int. J. Adhes. Adhes..

[22] Achilias D.S., Karabela M.M., Varkopoulou E.A., Sideridou I.D. (2012). Cure kinetics study of two epoxy systems with fourier tranform infrared spectroscopy (FTIR) and differential scanning calorimetry (DSC). J. Macromol. Sci. Part A.

[23] Prime R.B., Bair H.E., Vyazovkin S., Gallagher P.K., Riga A., Menczel J.D., Prime R.B. (2009). Thermogravimetric analysis (TGA). Thermal Analysis of Polymers: Fundamentals and Applications.

[24] Senturia S.D., Sheppard N.F. (2005). Epoxy Resins and Composites IV.

[25] Fraga F., Vazquez E.C., Rodríguez-Núñez E., Martínez-Ageitos J.M. (2008). Curing kinetics of the epoxy system diglycidyl ether of bisphenol A/isophoronediamine by Fourier transform infrared spectroscopy. Polym. Adv. Technol..

[26] Rothenhäusler F., Kettenbach M., Ruckdaeschel H. (2023). Influence of the Stoichiometric Ratio on the Curing Kinetics and Mechanical Properties of Epoxy Resin Cured with a Rosin-Based Anhydride. Macromol. Mater. Eng..

[27] Gupta N., Mahendran A.R., Weiss S., Khalifa M. (2024). Thermal curing behavior of phenol formaldehyde resin-impregnated paper evaluated using DSC and dielectric analysis. J. Therm. Anal. Calorim..

[28] Okeola A.A., Hernandez-Limon J.E., Tatar J. (2024). Core-Shell Rubber Nanoparticle-Modified CFRP/Steel Ambient-Cured Adhesive Joints: Curing Kinetics and Mechanical Behavior. Materials.

[29] El-Aouni N., Dagdag O., Haldhar R., Kim S.-C., Azogagh M., Berisha A., Sherif E.-S.M., Hsissou R., Elbachiri A., Ebenso E.E. (2023). One-pot synthesis of epoxy resin composite: Thermal, rheological and Monte Carlo investigations. Iran. Polym. J..

[30] Deriszadeh A., Shahraki F., Mostafa L., Ali A.B.M., Mohebbi-Kalhori D., Salahshour S., Alizad A. (2024). Epoxy/phenolic nanocomposite based adhesives: Non-isothermal cure kinetic study. Results Eng..

[31] Şengül B., Tüzün F.N. (2023). Investigation and assessment of epoxy resin adhesives using Fourier-transform infrared spectrometry. Mater. Werkst..

[32] Zhang F., Zhang L., Guo X., Cai Z., Huang K. (2023). Study on curing kinetics of epoxy system and DFT simulation. J. Therm. Anal. Calorim..

[33] Duller M., Mahendran A.R., Zikulnig-Rusch E.M. (2024). Investigating the thermal cure behavior of sorbitol-derived biobased melamine–formaldehyde impregnation resins using DSC and FTIR analysis. J. Therm. Anal. Calorim..

[34] Jouenne J.-B., Barbier D., Hounkpati V., Cauret L., Vivet A. (2024). Influence of flax fibers on the curing kinetics of bio-based epoxy resin. J. Mater. Sci..

[35] YÜKsel O., Yildirim E., YÜCel O., EmİK S. (2023). Synthesis and Investigation of Thermal and Dynamic Mechanical Properties of Urethane-Containing Epoxy Resins. J. Turk. Chem. Soc. Sect. B Chem. Eng..

[36] Singh S.K., Kandpal S., Rajpoot M.S., Chandra P. (2024). Investigating the Mechanical and Thermal Properties of PDMS-Toughened Epoxy Resins for Advanced Adhesive Solutions. J. Mater. Sci. Chem. Eng..

[37] Aliakbari M., Moini Jazani O., Moghadam M., Martín-Martínez J.M. (2024). Manipulating a novel epoxy-based composite with core–shell rubber particles for designing a structural adhesive in aluminum–aluminum bonded joints. Polym. Adv. Technol..

[38] Wang Y., Nian G., Yang X., Suo Z. (2021). Lap shear of a soft and elastic adhesive. Mech. Mater..

[39] Cui J., Wang S., Wang S., Chen S., Li G. (2020). Strength and failure analysis of adhesive single-lap joints under shear loading: Effects of surface morphologies and overlap zone parameters. J. Manuf. Process..

[40] Naat N., Boutar Y., Naïmi S., Mezlini S., Da Silva L.F.M. (2023). Effect of surface texture on the mechanical performance of bonded joints: A review. J. Adhes..

[41] Kanani A.Y., Hou X., Laidlaw R., Ye J. (2021). The effect of joint configuration on the strength and stress distributions of dissimilar adhesively bonded joints. Eng. Struct..

[42] Sousa F.C., Akhavan-Safar A., Carbas R.J.C., Marques E.A.S., Goyal R., Jennings J., da Silva L.F.M. (2024). Investigation of geometric and material effects on the fatigue performance of composite and steel adhesive joints. Compos. Struct..

[43] Guo L., Liu J., Xia H., Li X., Zhang X., Yang H., Yang Y. (2022). Effects of loading rate, temperature, and thickness on the tensile strength of precision adhesive joints. Polym. Test..

[44] dos Reis M.Q., Marques E.A.S., Carbas R.J.C., da Silva L.F.M. (2020). Functionally graded adherends in adhesive joints: An overview. J. Adv. Join. Process..

[45] Marques J.B., Barbosa A.Q., da Silva C.I., Carbas R.J.C., da Silva L.F.M. (2019). An overview of manufacturing functionally graded adhesives–Challenges and prospects. J. Adhes..

[46] Boulenouar A., Bouchelarm M.A., Chafi M. (2024). Numerical investigation of cracked metal/ceramic FGM plates repaired with bonded composite patch. Int. J. Interact. Des. Manuf. (IJIDeM).

[47] Benali A., Cellard C., Sohier L., Moretti A., Créac’hcadec R. (2023). Modelling edge effects at the interface in bonded joints using gradient functions in the mechanical properties of the adhesive: Application of the method to the Arcan test loaded in tension and shear. Int. J. Adhes. Adhes..

[48] Jia Z., Yu J., Liu Q., Yu S., Wang Z. (2023). Functionally graded adhesive joints with exceptional strength and toughness by graphene nanoplatelets reinforced epoxy adhesives. Int. J. Adhes. Adhes..

[49] Ferreira C., Campilho R., Moreira R. (2020). Bonded structures improvement by the dual adhesive technique. Procedia Struct. Integr..

[50] Khan M.A., Tipireddy R., Dattaguru B., Kumar S. (2023). Stochastic modeling of functionally graded double lap adhesive joints. Mech. Mater..

[51] Akhavan-Safar A., Ramezani F., Delzendehrooy F., Ayatollahi M.R., da Silva L.F.M. (2022). A review on bi-adhesive joints: Benefits and challenges. Int. J. Adhes. Adhes..

[52] da Silva C.I., Cunha M.R.O., Barbosa A.Q., Carbas R.J.C., Marques E.A.S., da Silva L.F.M. (2021). Functionally graded adhesive joints using magnetic microparticles with a polyurethane adhesive. J. Adv. Join. Process..

[53] Ramalho L.D.C., Campilho R.D.S.G., Belinha J., da Silva L.F.M. (2020). Static strength prediction of adhesive joints: A review. Int. J. Adhes. Adhes..

[54] Hasheminia S.M., Park B.C., Chun H.-J., Park J.-C., Chang H.S. (2019). Failure mechanism of bonded joints with similar and dissimilar material. Compos. Part B Eng..

[55] Kim M.-H., Hong H.-S., Kim Y.-C. (2021). Determination of failure envelope of functionally graded adhesive bonded joints by using mixed mode continuum damage model and response surface method. Int. J. Adhes. Adhes..

[56] Nimje S.V., Panigrahi S.K., Mittal K.L., Panigrahi S.K. (2020). Damage behaviour in functionally graded structural adhesive joints with double lap joint configuration. Structural Adhesive Joints.

[57] Dadian A., Rahnama S. (2021). Experimental and numerical study of optimum functionally graded Aluminum/GFRP adhesive lap shear joints using Epoxy/CTBN. Int. J. Adhes. Adhes..

[58] Li R., Noda N.-A., Takaki R., Sano Y., Takase Y., Miyazaki T. (2018). Most suitable evaluation method for adhesive strength to minimize bend effect in lap joints in terms of the intensity of singular stress field. Int. J. Adhes. Adhes..

[59] Sika Hellas (2020). Product Data Sheet: SikaPower^®^-1511. https://www.google.com/url?sa=t&source=web&rct=j&opi=89978449&url=https://grc.sika.com/dms/getdocument.get/435e3071-7a94-43be-ad05-8e30be6f6020/013106155110001000_SikaPower_1511_eng.pdf&ved=2ahUKEwj9qcOp6bOKAxUiVqQEHX8BKvoQFnoECBgQAQ&usg=AOvVaw0TMBeaDYUIqOP3nQMaDZIG.

[60] Sika Hellas (2019). Product Data Sheet: SikaPower®-1548. https://www.google.com/url?sa=t&source=web&rct=j&opi=89978449&url=https://ita.sika.com/dms/getdocument.get/7df29c35-3696-4966-b08a-816759f38da5/sikapower_-1548.pdf&ved=2ahUKEwj0xMTz6bOKAxWHTaQEHXMNHK0QFnoECBwQAQ&usg=AOvVaw11Aah0oPy0BpnfuoHK3D3Q.

[61] Vyazovkin S., Wight C.A. (1998). Isothermal and non-isothermal kinetics of thermally stimulated reactions of solids. Int. Rev. Phys. Chem..

[62] Barbosa A.Q., da Silva L.F.M., Abenojar J., Figueiredo M., Öchsner A. (2017). Toughness of a brittle epoxy resin reinforced with micro cork particles: Effect of size, amount and surface treatment. Compos. Part B Eng..

[63] (2020). Plastics-Determination of Tensile Properties-Part 1: General Principles.

[64] (2021). Standard Test Method for Rubber Property—Durometer Hardness.

[65] da Silva L.F.M., Rodrigues T.N.S.S., Figueiredo M.A.V., de Moura M.F.S.F., Chousal J.A.G. (2006). Effect of Adhesive Type and Thickness on the Lap Shear Strength. J. Adhes..

[66] Socrates G. (2004). Infrared and Raman Characteristic Group Frequencies: Tables and Charts.

[67] Saleh T.A., Saleh T.A. (2022). Surface Science of Adsorbents and Nanoadsorbents. Interface Science and Technology.

[68] Noda N.-A., Chen D., Zhang G., Sano Y. (2020). Single-fiber pull-out analysis comparing the intensities of singular stress fields (ISSFs) at fiber end/entry points. Int. J. Mol. Sci..

[69] Noda N.-A., Takaki R., Sano Y., Wang B. (2023). ISSF method to evaluate adhesive strength when two distinct singular stress fields appear along the interface. Indones. J. For. Res..

[70] Dundurs J. (1969). Discussion:“Edge-bonded dissimilar orthogonal elastic wedges under normal and shear loading”(Bogy, DB, 1968, ASME J. Appl. Mech., 35, pp. 460–466). J. Appl. Mech..

[71] Noda N.-A., Miyazaki T., Li R., Uchikoba T., Sano Y., Takase Y. (2015). Debonding strength evaluation in terms of the intensity of singular stress at the interface corner with and without fictitious crack. Int. J. Adhes. Adhes..

[72] Noda N.-A., Li R., Miyazaki T., Takaki R., Sano Y. (2019). Convenient adhesive strength evaluation method in terms of the intensity of singular stress field. Int. J. Comput. Methods.

[73] Kamal M., Sourour S. (1973). Kinetics and thermal characterization of thermoset cure. Polym. Eng. Sci..

[74] Kissinger H.E. (1957). Reaction kinetics in differential thermal analysis. Anal. Chem..

[75] Vyazovkin S., Wight C.A. (1999). Model-free and model-fitting approaches to kinetic analysis of isothermal and nonisothermal data. Thermochim. Acta.

[76] Abenojar J., Enciso B., Pantoja M., Velasco F., Martínez M.-A. (2020). Thermal characterization and diffusivity of two mono-component epoxies for transformer insulation. Int. J. Adhes. Adhes..

[77] Abenojar J., Lopez de Armentia S., Barbosa A.Q., Martinez M.A., del Real J.C., da Silva L.F.M., Velasco F. (2022). Magnetic cork particles as reinforcement in an epoxy resin: Effect of size and amount on thermal properties. J. Therm. Anal. Calorim..

[78] Abenojar J., Tutor J., Ballesteros Y., del Real J.C., Martínez M.A. (2017). Erosion-wear, mechanical and thermal properties of silica filled epoxy nanocomposites. Compos. Part B Eng..

[79] Polymer Innovation Blog: Epoxy Curing Agents–Mercaptans, The Ultimate Quick Ambient Cure. https://polymerinnovationblog.com/epoxy-curing-agents-mercaptans-the-ultimate-quick-ambient-cure/.

[80] Tsuji Y. (2024). Molecular Understanding of the Distinction between Adhesive Failure and Cohesive Failure in Adhesive Bonds with Epoxy Resin Adhesives. Langmuir.

[81] Miyata T., Sato Y.K., Kawagoe Y., Shirasu K., Wang H.F., Kumagai A., Kinoshita S., Mizukami M., Yoshida K., Huang H.H. (2024). Effect of inorganic material surface chemistry on structures and fracture behaviours of epoxy resin. Nat. Commun..

[82] Jairaja R., Naik G.N. (2019). Single and dual adhesive bond strength analysis of single lap joint between dissimilar adherends. Int. J. Adhes. Adhes..

